# Multimodal prognosis of negative symptom severity in individuals at increased risk of developing psychosis

**DOI:** 10.1038/s41398-021-01409-4

**Published:** 2021-05-24

**Authors:** Daniel J. Hauke, André Schmidt, Erich Studerus, Christina Andreou, Anita Riecher-Rössler, Joaquim Radua, Joseph Kambeitz, Anne Ruef, Dominic B. Dwyer, Lana Kambeitz-Ilankovic, Theresa Lichtenstein, Rachele Sanfelici, Nora Penzel, Shalaila S. Haas, Linda A. Antonucci, Paris Alexandros Lalousis, Katharine Chisholm, Frauke Schultze-Lutter, Stephan Ruhrmann, Jarmo Hietala, Paolo Brambilla, Nikolaos Koutsouleris, Eva Meisenzahl, Christos Pantelis, Marlene Rosen, Raimo K. R. Salokangas, Rachel Upthegrove, Stephen J. Wood, Stefan Borgwardt, and the PRONIA Group

**Affiliations:** 1 Department of Psychiatry (UPK), University of Basel, Basel, Switzerland; 2 Department of Mathematics and Computer Science, University of Basel, Basel, Switzerland; 3 Department of Psychology, University of Basel, Basel, Switzerland; 4 Department of Psychiatry and Psychotherapy, University of Lübeck, Lübeck, Germany; 5 Imaging of Mood- and Anxiety-Related Disorders (IMARD) Group, Institut d’Investigacions Biomèdiques August Pi i Sunyer (IDIBAPS), CIBERSAM, Barcelona, Spain; 6 Early Psychosis: Interventions and Clinical-Detection (EPIC) Lab, Department of Psychosis Studies, Institute of Psychiatry, Psychology and Neuroscience, King’s College London, London, UK; 7 Department of Clinical Neuroscience, Karolinska Institute, Stockholm, Sweden; 8 Department of Psychiatry and Psychotherapy, Faculty of Medicine and University Hospital, University of Cologne, Cologne, Germany; 9 Department of Psychiatry and Psychotherapy, Ludwig-Maximilian-University, Munich, Germany; 10 Max Planck School of Cognition, Leipzig, Germany; 11 Department of Psychiatry, Icahn School of Medicine at Mount Sinai, New York, NY USA; 12 Department of Education, Psychology, Communication, University of Bari Aldo Moro, Bari, Italy; 13 Institute for Mental Health and Centre for Human Brain Health, University of Birmingham, Birmingham, UK; 14 Institute for Mental Health, University of Birmingham, Birmingham, UK; 15 Department of Psychiatry and Psychotherapy, Medical Faculty, Heinrich-Heine University, Düsseldorf, Germany; 16 University Hospital of Child and Adolescent Psychiatry and Psychotherapy, University of Bern, Bern, Switzerland; 17 Department of Psychology and Mental Health, Faculty of Psychology, Airlangga University, Surabaya, Indonesia; 18 Department of Psychiatry, University of Turku, Turku, Finland; 19 Department of Neurosciences and Mental Health, Fondazione IRCCS Ca’ Granda Ospedale Maggiore Policlinico, Milan, Italy; 20 Department of Pathophysiology and Transplantation, University of Milan, Milan, Italy; 21 Melbourne Neuropsychiatry Centre, University of Melbourne & Melbourne Health, Carlton South, VIC Australia; 22 Institute for Mental Health and School of Psychology, University of Birmingham, Birmingham, UK; 23 Orygen, Melbourne, VIC Australia; 24 Centre for Youth Mental Health, University of Melbourne, Melbourne, VIC Australia

**Keywords:** Schizophrenia, Neuroscience

## Abstract

Negative symptoms occur frequently in individuals at clinical high risk (CHR) for psychosis and contribute to functional impairments. The aim of this study was to predict negative symptom severity in CHR after 9 months. Predictive models either included baseline negative symptoms measured with the Structured Interview for Psychosis-Risk Syndromes (SIPS-N), whole-brain gyrification, or both to forecast negative symptoms of at least moderate severity in 94 CHR. We also conducted sequential risk stratification to stratify CHR into different risk groups based on the SIPS-N and gyrification model. Additionally, we assessed the models’ ability to predict functional outcomes in CHR and their transdiagnostic generalizability to predict negative symptoms in 96 patients with recent-onset psychosis (ROP) and 97 patients with recent-onset depression (ROD). Baseline SIPS-N and gyrification predicted moderate/severe negative symptoms with significant balanced accuracies of 68 and 62%, while the combined model achieved 73% accuracy. Sequential risk stratification stratified CHR into a high (83%), medium (40–64%), and low (19%) risk group regarding their risk of having moderate/severe negative symptoms at 9 months follow-up. The baseline SIPS-N model was also able to predict social (61%), but not role functioning (59%) at above-chance accuracies, whereas the gyrification model achieved significant accuracies in predicting both social (76%) and role (74%) functioning in CHR. Finally, only the baseline SIPS-N model showed transdiagnostic generalization to ROP (63%). This study delivers a multimodal prognostic model to identify those CHR with a clinically relevant negative symptom severity and functional impairments, potentially requiring further therapeutic consideration.

## Introduction

Precise prognosis of clinical outcomes in individuals at clinical high risk (CHR) of developing psychosis is imperative to guide treatment selection. Prognostic risk stratification models help to decide who will benefit most from active treatment^1^. While much effort has been dedicated to predicting transition to psychosis^2,3^, prognostic models focusing on negative symptom outcomes in CHR are missing. This is a major oversight, bearing in mind that 82% of CHR exhibit at least one negative symptom in the moderate to severe range at first clinical presentation, and 54% still meet this criterion after 12 months^4^. Negative symptoms are also strong predictors of poor functioning^5,6^ irrespective of other symptoms such as depression or anxiety^7^. Prognostic tools are therefore urgently required to track negative symptom progression in CHR and to identify those who might benefit most from potential interventions, such as *N*-methyl-d-asparate-receptor (NMDAR) modulators (in conjunction with psychosocial interventions^8^).

A key challenge in management of psychotic disorders is that clinical outcomes are difficult to prognosticate based on behavioural signs^9^. To overcome this issue, the field is searching for biologically informed prognostic assays^10^ to complement clinical information for increased treatment precision and/or prognostic indication^11,12^. Sequentially adding biological evidence to initial clinical assessments may help establish more accurate stratification models to forecast clinical outcomes in CHR^13,14^. Considering psychosis as a brain disorder^1,15^, neuroimaging offers a powerful tool to map pathophysiological processes associated with illness onset. However, most neuroimaging studies to date have reported differences at the group level, rendering personalized clinical decision-making difficult. Machine-learning provides a promising tool to address this issue^16,17^. For instance, a recent machine-learning study in CHR found that grey matter volume and clinical data could predict individual social functioning with more than 75% accuracy, while a combination of models further improved prognostic performance to 82%^18^. Furthermore, using surface-based measures like gyrification and surface area, other CHR studies showed that transition to psychosis or global functioning can be predicted with more than 80% accuracy^19,20^. The measurement of gyrification might be particularly sensitive to detect pathophysiology in the prodromal phase^21^. Gyrification is critical during early brain maturation^22,23^. Early maldevelopment may result in an intransient risk factor for emerging psychosis. This aligns with the developmental risk model of psychosis^24^, which integrates perinatal hazards and neurodevelopmental abnormalities with stressful experiences during adolescence into the pathogenesis of psychosis.

Here, we applied machine-learning^3,17,25^ to multisite data^18^ to predict negative symptoms of clinically relevant severity after 9 months using baseline negative symptoms measured with the Structured Interview for Psychosis-Risk Syndromes^26^ (SIPS-N), gyrification data, and their combination as predictors. Second, we conducted a two-step simulation approach^13,14^ by sequentially adding the gyrification model on top of the baseline SIPS-N model to stratify individuals into different risk groups. Third, bearing in mind that negative symptoms were related to poor functioning in CHR^5,6^, we assessed the ability of the three negative symptom models to predict functional impairments. Finally, we investigated transdiagnostic generalizability by employing the models trained in CHR to predict negative symptom severity in recent-onset psychosis (ROP) and recent-onset depression (ROD) patients. This last analysis is grounded in evidence showing an overlap between negative and the depressive symptoms^27^, pluripotent, transdiagnostic trajectories of CHR including non-psychotic disorders^11,28^, and risk-associated disruption of brain circuits that may mediate susceptibility to broad domains of psychopathology rather than discrete disorders^29^. This holistic strategy is useful for predicting clinical outcomes transdiagnostically, paving the way for the development of transdiagnostic, preventative interventions.

## Subjects and methods

### Subjects

As part of a European multisite study, the ‘Personalized Prognostic Tools for Early Psychosis Management’ (PRONIA; http://www.pronia.eu; see ref. ^18^ for more details on project design), CHR, ROP, and ROD patients were recruited at seven sites in five countries. Only individuals with complete negative symptom and gyrification data at baseline and negative symptom assessment at the 9-month assessment were included yielding 94 CHR, 96 ROP, and 97 ROD. Written informed consent was obtained from all participants. The authors assert that all procedures contributing to this work comply with the ethical standards of the relevant national and institutional committees on human experimentation and with the Helsinki Declaration of 1975, as revised in 2008. All procedures involving human participants were approved by the respective local ethics committee.

General inclusion criteria were age between 15 and 40 years, sufficient language skills for participation, as well as capacity to provide informed consent. General exclusion criteria were an IQ below 70, current or past head trauma with loss of consciousness (>5 min), current or past known neurological or somatic disorders, current or past alcohol dependence, or polysubstance dependence within the past 6 months, cannabis consumption in the last month and any medical indication against MRI. The CHR state was defined by either (a) cognitive disturbances (COGDIS) criteria assessed using the Schizophrenia Proneness Instrument (SPI-A)^30^ (see refs. ^31,32^ for more details) and/or (b) ultra-high-risk criteria for psychosis based on the Structured Interview for Psychosis-Risk Syndromes (SIPS^26^) (see refs. ^32,33^ for more details). Exclusion criteria for CHR were (i) antipsychotic medication for >30 days (cumulative number of days) at or above minimum dosage of the ‘1st episode psychosis’ range of DGPPN S3 guidelines^34^ and (ii) any intake of antipsychotic medication within the past 3 months before clinical baseline assessments at or above minimum dosage of the ‘1st episode psychosis’ range of DGPPN S3 guidelines^34^. Detailed in- and exclusion criteria for the ROP and ROD groups can be found in the supplement.

### Clinical assessment

All participants underwent clinical assessment of positive, negative, disorganized, and general symptoms using the SIPS^26^. Furthermore, depressive symptoms were assessed with Beck’s Depression Inventory (BDI)^35^, and social and role functioning with the Global Functioning: Social and Role scales^36^.

### Neuroimaging assessment

Gyrification was derived from a T1-weighted structural MRI. (See Table S1 for data acquisition parameters). All images were processed and analysed using the Computational Anatomy Toolbox (CAT12; version r1155; http://dbm.neuro.uni-jena.de/cat12/), an extension toolbox of Statistical Parametric Mapping software (SPM12, http://www.fil.ion.ucl.ac.uk/spm/software/spm12). Gyrification values were then extracted for 68 regions (34 for each hemisphere) using the Desikan–Killiany atlas^37^ as implemented in CAT12 and normalized for individual intracranial volumes given that gyrification may correlate with the brain volume^38^. More detailed information is provided in the Supplement.

### Outcome definition

#### Negative symptom outcomes

According to Cornblatt and colleagues^4,39–41^, individuals with a score of ≥3 in any of the SIPS negative symptom items can be considered as having moderate to severe negative symptoms. Scores equal to or greater than 3 on the SIPS have been rated as clinically significant^42^ and 54% of CHR still exhibit such symptoms after 12 months^4^. Notably, SIPS negative symptom items are more severe and persistent in individuals who convert to psychosis^4^ and longer duration and severity of SIPS negative symptom items are related to poor social functioning^41^. The outcome label was defined using N1 (social anhedonia), N2 (avolition), N3 (expression of emotion), N4 (experience of emotions and self) and N6 (occupational functioning); we excluded N5 (ideational richness) because several factor analyses found N5 to be either unrelated to the other negative symptoms^43,44^ or only weakly related to them when they were exclusively examined^45^. The latter factor analysis further indicated a two-factor model with two SIPS negative symptom dimensions reflecting *volition* (avolition and occupational functioning) and *emotion* (expression and experience of emotion and social anhedonia)^45^. While the volition factors showed an association with poor role function, the emotion factor was associated with poor social function.

#### Functional outcomes

In line with a recent analysis^18^, we used the Global Functioning: Social and Role scales^36^ to define adequate (more than 7 points) vs impaired (7 or fewer points) social and role functioning at a 9-month follow-up assessment.

### Predictor variables

#### Baseline SIPS-N model

The baseline SIPS-N model used individual SIPS baseline items N1, N2, N3, N4, and N6 as predictors.

#### Gyrification model

The gyrification model used individual gyrification values for all 68 regions as predictors.

### Statistical analysis

#### Machine-learning analysis to predict negative symptom severity

We trained three different models to predict negative symptom outcomes in CHR using the NeuroMiner software package (version: 1.0; release: elessar; https://www.pronia.eu/neurominer) in Matlab (release: R2017a; https://de.mathworks.com/products/matlab.html). The first two models were trained using baseline SIPS-N and gyrification data as predictors, respectively (see above). The third model constituted a stacking model that combined predictions of the SIPS-N and gyrification model. Methodological details of model construction can be found in the supplement. We used nested cross-validation with a leave-site-out cross-validation (LSO-CV) in the outer loop to assess geographic generalizability to a hypothetical new centre and fivefold repeated cross-validation with 10 repetitions in the inner loop. To assess whether models achieved above-chance performance, we computed the posterior distributions of the balanced accuracy (BAC)^46^ and considered the performance to be significant, if 95% of the posterior mass fell above a BAC of 0.5, as 0.5 indicates chance level in a binary classification context. Note that we report the LSO-CV point estimate, but also computed other moments of the posterior distribution and positive and negative predictive values (PPV/NPV)). To further investigate which predictors reliably drove the classification performance of the models, we computed cross-validation ratio profiles (CVR = mean(*w*)/SE(*w*), where *w* corresponds to the normalized weight vector under Euclidian assumptions of the linear classifier (see ref. ^18^ for more details).

#### Sequential risk stratification

In real-world clinical settings clinical information is usually collected sequentially to minimize the burden for patients and healthcare costs, rather than simultaneously as in our study. To simulate this scenario, we performed a sequential risk stratification based on the base rate of experiencing moderate/severe negative symptoms and the sensitivity and specificity of the baseline SIPS-N and gyrification model derived from our sample. The goal of this analysis was to stratify CHR into different risk groups to guide future treatment selection by estimating the theoretical PPV of a two-stage probabilistic assessment approach (see refs. ^13,14^ for technical details). In brief, starting with the pretest probability of having moderate/severe negative symptoms at follow-up in our own CHR sample (40%), we simulated a hypothetical scenario in which each individual would be subjected first to a clinical baseline assessment using the SIPS negative items and subsequently undergo a second test, here a structural MRI, from which a gyrification signature would be computed. Following this procedure, we were able to stratify individuals into different risk groups with respect to the probability of presenting moderate/severe negative symptoms at follow-up. Based on the risk ratio of a recent clinical pilot trial in CHR^47^, we finally assessed the theoretical clinical efficacy of this two-stage sequential testing approach by estimating the number needed to treat (NNT) for each risk group to achieve remission of negative symptoms when undergoing a d-serine treatment (for a schematic analysis overview see Fig. S1).

#### Assessing outcome generalization of negative symptom models to functional outcomes in CHR

Next, we assessed whether the models that were trained on baseline data to predict negative symptoms in CHR were also able to predict adequate vs impaired social and role functioning at follow-up in the same group.

#### Assessing transdiagnostic generalization of negative symptom models to other patient populations

To test transdiagnostic generalizability of models, we applied the negative symptom models trained in the CHR sample to patients with ROP and ROD.

## Results and discussion

### Results

#### Demographic and clinical features

Demographics and clinical characteristic of the whole study sample are described in Table 1, S2, and S3. In the CHR group, 38 individuals (40%) presented at least one negative symptom rated ≥3 on the SIPS (i.e. moderate/severe severity) at follow-up, while 56 patients (60%) displayed mild or no negative symptoms (scores ≤ 2). The groups did not differ in age, sex, handedness, and education. The moderate/severe negative symptom group showed higher negative symptoms and lower social/role functioning at baseline and follow-up (see Fig. S2). To assess whether negative symptom outcomes were confounded by secondary negative symptoms^48^, we computed correlations between outcomes, and SIPS positive as well as BDI depressive symptoms at baseline. This analysis suggested that negative symptoms at follow-up could not be explained by secondary negative symptoms resulting from positive (*r* = −0.069, *p* = 1.000) or depressive symptoms (*r* = −0.075, *p* = 0.941).

**Table 1:** Clinical and demographic characteristics of the study sample.

	Clinical high risk for psychosis (CHR)			Recent-onset psychosis (ROP)			Recent-onset depression (ROD)		
Moderate/severe negative symptoms at T1	Yes	No	Statistic	Yes	No	Statistic	Yes	No	Statistic
Total number participants (%)	38 (40)	56 (60)		57 (59)	39 (41)		31 (32)	66 (68)	
Age, mean (SD)^a^	23.75 (4.71)	24.49 (5.69)	*t*_92_ = −0.689, *p* = 0.492	24.79 (4.94)	26.10 (6.08)	*t*_94_ = −1.088, *p* = 0.280	26.69 (6.48)	26.76 (6.00)	*t*_95_ = −0.055, *p* = 0.956
Sex, women/men^b^	15/23	29/27	*χ*^2^_1_ = 1.378, *p* = 0.240	16/41	19/20	*χ*^2^_1_ = 4.261, ***p*** = **0.039**	15/16	40/26	*χ*^2^_6_ = 1.283, *p* = 0.257
Years of education, mean (SD)^a^	13.17 (2.88)	14.02 (3.18)	*t*_91_ = −1.336, *p* = 0.185	13.34 (2.92)	15.13 (3.46)	*t*_94_ = −2.656, ***p*** = **0.010**	14.65 (3.00)	15.10 (2.97)	*t*_94_ = −0.696, *p* = 0.489
SIPS negative symptoms T0, median [25th percentile, 75th percentile]^c^	13.50_*n*=38_ [5.75, 6.25]	7.00_*n*=56_ [3.00, 10.00]	*U* = 610.000, ***p*** < **0.001**	12.00 _*n*=57_ [6.75, 17.00]	7.50 _*n*=39_ [3.75, 11.00]	*U* = 750.500, ***p*** = **0.007**	9.50 _*n*=31_ [6.25, 13.75]	7.00 _*n*=65_ [4.00, 11.00]	*U* = 754.000, ***p*** = **0.047**
SIPS negative symptoms T1, median [25th percentile, 75th percentile]^c^	7.50 _*n*=38_ [6.75, 10.00]	1.00 _*n*=56_ [0.00, 3.00]	*U* = 33.500, ***p*** < **0.001**	9.00 _*n*=57_ [6.00, 14.00]	0.00 _*n*=39_ [0.00, 2.25]	*U* = 90.000, ***p*** < **0.001**	8.00 _*n*=31_ [5.25, 10.00]	0.50 _*n*=66_ [0.00, 5.00]	*U* = 86.000, ***p*** < **0.001**
Global functioning: Social T0, median [25th percentile, 75th percentile]^c^	6.00_*n*=38_ [5.00, 7.00]	7.00_*n*=56_ [6.00, 8.00]	*U* = 507.500, ***p*** < **0.001**	5.50 _*n*=57_ [5.00, 7.00]	6.00 _*n*=39_ [5.00, 7.00]	*U* = 779.000, ***p*** = **0.011**	6.00 _*n*=31_ [5.00, 7.00]	7.00 _n=65_ [6.00, 8.00]	*U* = 719.000, ***p*** = **0.020**
Global functioning: Social T1, median [25th percentile, 75th percentile]^c^	6.00 _*n*=37_ [6.00, 7.00]	8.00 _*n*=56_ [7.00, 8.00]	*U* = 356.000, ***p*** < **0.001**	7.00 _*n*=56_ [5.00, 8.00]	8.00 _*n*=39_ [7.00, 8.00]	*U* = 668.000, ***p*** < **0.001**	6.50 _*n*=31_ [5.25, 8.00]	8.00 _*n*=66_ [7.00, 8.00]	*U* = 557.500, ***p*** < **0.001**
Global functioning: Role T0. median [25th percentile, 75th percentile]^c^	5.00 _*n*=38_ [5.00, 6.00]	7.00 _*n*=56_ [6.00, 8.00]	*U* = 498.000, ***p*** < **0.001**	5.00 _*n*=57_ [4.00, 6.00]	6.00 _*n*=39_ [5.00, 7.00]	*U* = 706.500, ***p*** = **0.002**	6.50 _*n*=31_ [5.25, 8.00]	7.00 _*n*=65_ [6.00, 7.00]	*U* = 948.500, *p* = 0.637
Global functioning: role T1 median [25th percentile, 75th percentile]^c^	6.00 _*n*=37_ [4.00, 7.00]	8.00_*n*=56_ [7.00, 8.00]	*U* = 278.500, ***P*** < **0.001**	6.00 _*n*=56_ [4.00, 7.00]	7.50 _*n*=39_ [6.00, 8.00]	*U* = 605.000, ***p*** < **0.001**	7.00 _*n*=31_ [6.00, 8.00]	8.00 _*n*=66_ [7.00, 8.00]	*U* = 531.000, ***p*** < **0.001**

*BDI* Becks depression inventory^35^, *SIPS* structured interview for prodromal syndromes^26^, *T0* baseline assessment, *T1* 9-month follow-up assessment. ^**a**^Two-sample *t*-tests between individuals with moderate/severe (any score ≥3) and mild (all scores <3) negative symptoms at follow-up. ^b^*χ*^2^-tests between individuals with moderate/severe (any score ≥3) and mild (all scores <3) negative symptoms at follow-up. ^c^Two-sample Mann–Whitney *U*-tests between individuals with moderate/severe (any score ≥3) and mild (all scores <3) negative symptoms at follow-up. Please refer to Table S3 for a more extensive overview.

Bold font indicates significant group differences at the conventional p < 0.05 threshold.

In the ROP group, 57 patients (59%) showed moderate/severe negative symptom outcomes, whereas 39 patients (41%) exhibited mild negative symptoms. These groups did not differ in age, handedness, but in sex and education. ROP patients with moderate/severe negative symptom outcomes at follow-up presented higher negative symptoms and lower social/role functioning at baseline and follow-up.

In the ROD group, 31 patients (32%) suffered from moderate/severe negative symptoms at follow-up, while 66 patients (68%) showed mild negative symptoms. These two groups did not differ in age, sex, handedness, and education. Similar to the other groups moderate/severe negative symptom outcomes were associated with higher negative symptoms and poorer social, but not role functioning at baseline and higher negative symptoms as well as poorer social/role functioning at follow-up.

#### Multimodal prognosis of negative symptom severity in CHR patients

##### Baseline SIPS-N model

**Fig. 1:**
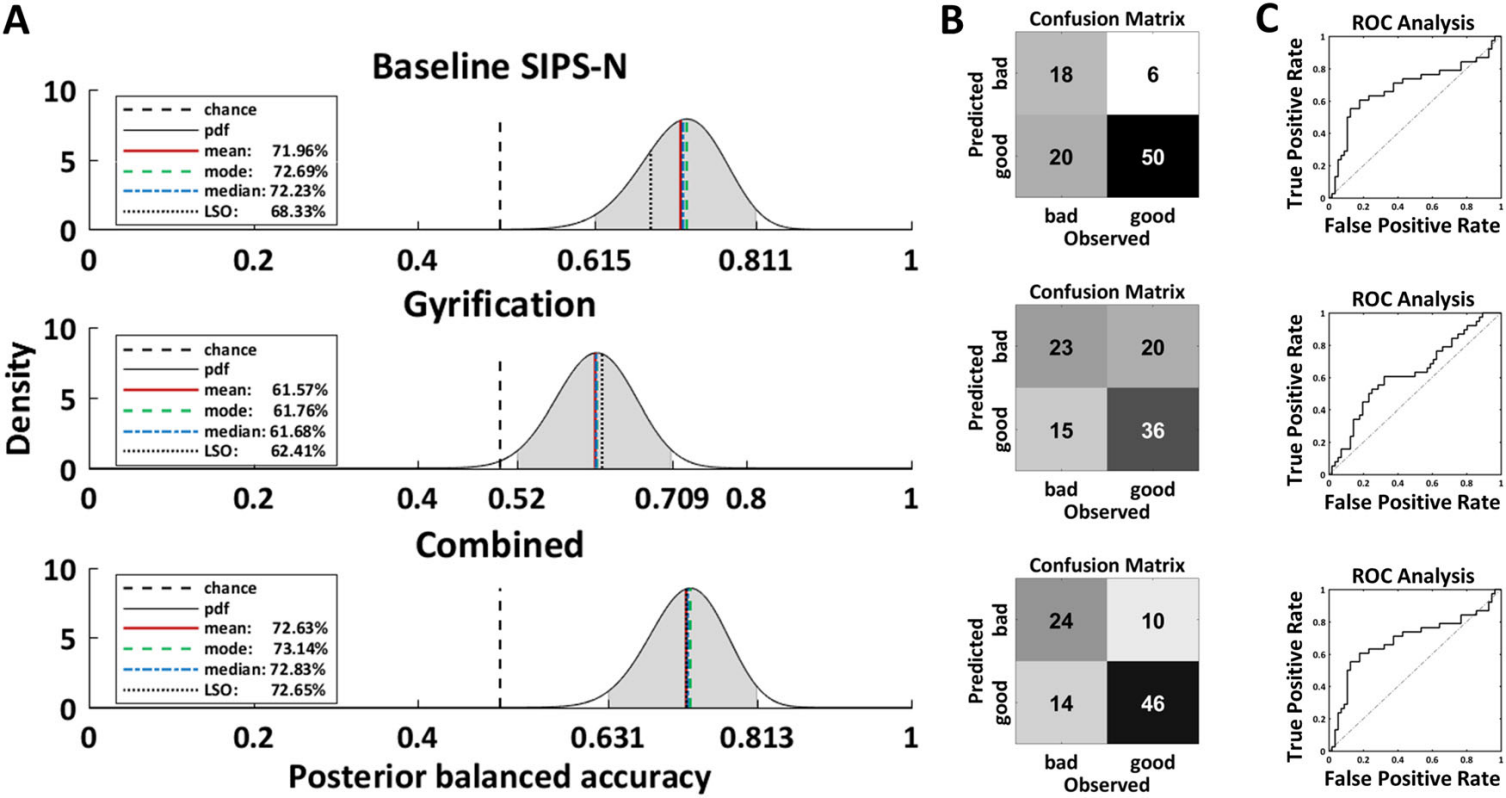
Model performances. **A** Posterior balanced accuracy distributions of baseline negative symptom (upper panel), gyrification (middle panel), and combined model (lower panel). Shaded grey area indicates 95% of the probability mass of the respective posterior distribution over the balanced accuracy. **B** Confusion matrices and **C** receiver operating characteristic (ROC) curves of the prediction models. *Bad outcome*: Expression of moderate to severe negative symptoms at follow-up (any score ≥3). *Good outcome*: All SIPS negative items <3 at follow-up. *SIPS-N* negative symptoms measured the Structured Interview for Psychosis-Risk Syndromes ^26^.

**Fig. 2:**
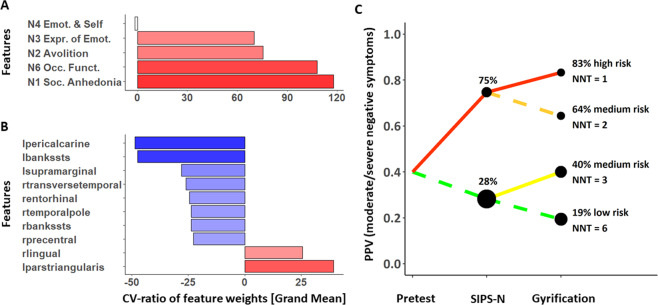
Feature importance. **A** Feature importance of baseline negative symptom model. Feature importance was measured through cross-validation (CV) ratio profiles (CVR = mean*(w)*/SE(*w)*, where *w* corresponds to the normalized weight vector under Euclidian assumptions of the logistic regression or support vector machine classifier; see ref. ^18^ for more details). Negative CVRs indicate that reduced values of the predictor are associated with increased risk of expressing negative symptoms, whereas positive values imply that an increase of the predictor value is associated with increased risk. **B** Top 10 most important features of the gyrification model. **C** Probabilistic assessment diagram illustrating two-stage sequential risk stratification to stratify patients based on their risk to develop moderate to severe negative symptoms. Note that this computation is based on (1) the base rate, as well as sensitivity and specificity from (2) the baseline SIPS-N and (3) the gyrification model, all derived from our sample. *X*-axis: Sequential tests (based on baseline negative symptoms and gyrification). *Y*-axis: Positive predictive value (PPV) associated with expressing moderate/severe negative symptoms at 9 months follow-up. The pretest probability was set to 40% based on our own sample (see Table 1). Each bifurcation in the plot represents the updated PPV after knowing that a test either yielded a positive (ascending solid line) or a negative result (descending dashed line). For a schematic analysis overview, please refer to Fig. S1. Line colour: Level of risk as previously suggested^14^. High**:** >80%, medium: 40–64%, and low: <20%. Dot sizes: Relative proportion of participants in our sample with a corresponding number of positive tests. The diagram also illustrates the number needed to treat (NNT) at each node, which is based on the risk ratio of a recent clinical pilot trial with a d-serine intervention to treat negative symptoms in clinical high-risk individuals^47^. *SIPS-N* negative symptoms measured the Structured Interview for Psychosis-Risk Syndromes^26^.

The baseline SIPS-N model achieved an above-chance BAC of 68% in predicting moderate/severe negative symptoms at follow-up in CHR (CI: [62%, 81%]; Fig. 1A and Table 2). Feature ranking showed that social anhedonia contributed most robustly to the classification, followed by occupational functioning and avolition (Fig. 2A).

**Table 2:** Classification performance, outcome generalization performance and transdiagnostic generalization performance.

	LSO-BAC (%)	AUC (%)	SE (%)	SP (%)	PPV (%)	NPV (%)
**Classification performance of prognostic models**						
Baseline SIPS-N model	68* [62, 81]	68	47	89	75	71
Gyrification model	62* [52, 71]	63	61	64	53	71
Combined model	73* [63, 81]	71	63	82	71	63
**Generalization to GF R outcome**						
Baseline SIPS-N model	59 [50, 70]	67	37	85	76	52
Gyrification model	74* [64, 81]	79	65	83	83	65
Combined model	63* [53, 72]	69	48	78	74	54
**Generalization to GF S outcome**						
Baseline SIPS-N model	61* [52, 72]	67	37	85	76	51
Gyrification model	76* [67, 83]	80	67	85	85	67
Combined model	65* [56, 74]	70	50	80	76	56
**Generalization to ROP**						
Baseline SIPS-N model	63* [53, 71]	63	51	74	74	51
Gyrification model	55 [45, 65]	53	35	74	67	44
Combined model	64* [54, 72]	63	67	62	72	56
**Generalization to ROD**						
Baseline SIPS-N model	57 [48, 70]	62	32	82	45	72
Gyrification model	48 [39, 57]	49	45	50	30	66
Combined model	60 [50, 68]	61	55	65	43	75

*SIPS-N* negative symptoms measured with the structured interview for prodromal syndromes^26^, *LSO-BAC* leave-site-out balanced accuracy point estimate, *AUC* area under the curve, *SE* sensitivity, *SP* specificity, *PPV* positive predictive value, *NPV* negative predictive value, *GF R* global functioning role outcome^36^, *GF S* global functioning social outcome^36^. Square brackets indicate lower and upper limit of 95% confidence interval around posterior balanced accuracy estimate. indicates significantly better than chance level (i.e., 50% not included in 95% of the probability mass of the posterior distribution over the model’s balanced accuracy).

Bold font indicates signficantly better than chance level (i.e.,50% not included in 95% of the probability mass of the posterior distribution over the model’s balanced accuracy).

##### Gyrification model

The gyrification model achieved a significant BAC of 62% (CI: [52%, 71%]; Fig. 1A and Table 2). Feature ranking showed that reduced gyrification in the left pericalcarine gyrus, left posterior superior temporal sulcus (pSTS), left supramarginal gyrus, right transverse temporal gyrus, and increased gyrification in the left inferior frontal gyrus (IFG) (pars triangularis) was associated with increased risk of expressing moderate/severe negative symptoms contributing most to the classification (Fig. 2B). Gyrification values for each region and group are reported in Table S4.

##### Combined model

Combining the baseline SIPS-N and gyrification model improved the BAC to 73% (CI: [63%, 81%]; Fig. 1A and Table 2).

##### Sequential risk stratification

Sequentially adding prognostic performance of the gyrification model on top of the baseline SIPS-N model led to a PPV of 83% (empirical probability in our sample: 91%) for an individual with two positive tests (high-risk group), 64 and 40% (empirical probabilities in our sample: 62 and 41%) for an individual with one positive and one negative test (medium-risk group) and 19% (empirical probabilities in our sample: 18%) for an individual with no positive tests (low-risk group; Fig. 2C). Accordingly, the hypothetical NNT for a d-serine intervention was 1 for those with two positive tests, 2–3 for those with one positive test, and 6 for those with no positive test (see Fig. 2C).

##### Outcome generalization of negative symptom models to functional outcomes in CHR

Social and role functioning data of one participant were missing. For the remaining 93 CHR, the baseline SIPS-N model produced an above-chance BAC for social (62%; CI: [52%, 72%], see Fig. S3A and Table 2), but not role functioning (59%; CI: [50%, 70%]). The gyrification model generalized to both role (74%; CI: [64%, 81%]) and social functioning (76%; CI: [67%, 83%]) with very high accuracy. Lastly, the combined model also predicted role (63%; CI: [54%, 73%]) and social functioning (64%; CI: [56%, 74%]) with intermediate BACs.

##### Transdiagnostic generalization of negative symptom models to other patient populations

The baseline SIPS-N model generalized to ROP (63%; CI: [53%, 71%], see Fig. S3B and Table 2), but not to ROD patients (57%; CI: [48%, 70%]). In contrast, the gyrification model failed to generalize to both ROP (55%; CI: [45%, 65%]) and ROD patients (48%; CI: [39%, 57%]). Lastly, the combined model generalized to ROP (64%; CI: [54%, 72%]), but not ROD patients (60%; CI: [50%, 68%]).

### Discussion

To the best of our knowledge this is the first study using state-of-the-art predictive modelling^49^ to forecast negative symptom severity in CHR after 9 months. Four major results were obtained: First, SIPS-N and gyrification baseline data predicted the presentation of moderate/severe negative symptom expression with an above-chance BAC of 68% and 62%, respectively, while the combined model achieved 73% BAC. Secondly, sequential testing allowed the stratification of CHR individuals into high (83%), medium (40–64%), and low (19%) groups regarding their risk to present moderate to severe negative symptoms at follow-up. Thirdly, we found that the SIPS-N model was also able to predict social (61%), but not role functioning at above-chance accuracy in CHR, whereas the gyrification model predicted both role (74%) and social (76%) functioning with high accuracies. The combined model also predicted role (63%) and social (65%) functioning. Finally, we found that the SIPS-N and the combined (63%, 64%), but not the gyrification model generalized to ROP, but not ROD patients for negative symptom prediction.

#### Baseline expression of negative symptoms predicts negative symptom severity at follow-up

We found that baseline expression of negative symptoms predicted moderate/severe negative symptoms in CHR with 68% BAC. Social anhedonia contributed most to the prognostic performance, followed by occupational functioning and avolition. This finding resonates with recent results showing that premorbid social maladjustment in late adolescence strongly predicted social anhedonia, and to a lesser extent occupational functioning, avolition, and expression of emotions in CHR^50^. It has been argued that a reciprocal relationship may exist between premorbid social adjustment and social anhedonia, possibly aggravating negative symptoms over time^51^. Our baseline SIPS-N model also achieved above-chance BAC in predicting impaired social functioning in CHR, generally supporting the inverse relationship between negative symptoms and social functioning in CHR^5^. It did not, however, generalize to role functioning. A recent factor analysis demonstrated that SIPS negative symptoms reflect two factors: (1) *volition* including avolition and occupational functioning and (2) *emotion* subsuming expression of emotion, social anhedonia, and experience of emotion^45^. The authors further reported that the emotion factor was associated with poor social function, whereas the volition factor was more related to poor role function^45^. Since social anhedonia (emotion factor) was weighted strongest in our model, it may be expected that the model generalizes better to social rather than role functioning. Lastly, we observed that the baseline SIPS-N model partially generalized to ROP patients, which may be explained by the large phenomenological overlap of the two populations. Interestingly, the baseline SIPS-N model did not generalize to ROD patients in our sample, possibly indicating specificity to the psychosis spectrum. This result resonates with evidence suggesting that at least some negative symptoms (e.g. social anhedonia) may be temporally linked to depressive episodes, whereas they appear to be more trait-like in the psychosis spectrum^52,53^. Another recent study found an association of the volition factor with subsyndromal depressive symptoms in bipolar I disorder, but not in schizophrenia^54^, highlighting the possibility that negative symptoms of similar severity may indeed be caused by distinct mechanisms in these two disorders.

#### Baseline gyrification predicts negative symptom severity at follow-up

Secondly, we found that whole-brain gyrification pattern also predicted moderate/severe negative symptoms in CHR with 62% BAC. In particular, we found that left pericalcarine gyrus, pSTS, left pars triangularis/IFG, left supramarginal gyrus, and right transverse temporal gyrus contributed most to the classification. In 80% of the top ten predictors, reduced gyrification was associated with increased risk of expressing moderate /severe negative symptoms, which is in line with previous evidence indicating that overall gyrification reductions are related to the severity of negative symptoms in patients with schizophrenia^55^. A number of these regions pertain to the ‘mentalizing network’^56^. Specifically, the pSTS has been implicated in perception of biological motion^57^, and gaze tracking^58^, but also, in conjunction with the temporoparietal junction, in implicit mentalizing^59^. The IFG has also been related to this network^60,61^, as mirror neurons have been identified there^62^ and lesions in this region lead to deficits in emotional empathy^63^. Predominance of these regions in our gyrification model may also explain why we found strong generalization performances to social and role functioning outcomes. Furthermore, the gyrification model did not generalize to ROD patients. These results resonate with other findings showing that the overlap changes in cortical gyrification in patients with schizophrenia and bipolar disorder is small (25%)^64^, and that there may be distinct mechanisms underlying negative symptoms in depression vs psychosis spectrum disorders, as alluded to above. Interestingly, the gyrification model did also not generalize to ROP, possibly indicating that developmental changes in gyrification provide a unique signature for the psychosis high-risk state.

#### Simultaneous and sequential multimodal prediction of negative symptom severity at follow-up

We observed a prognostic benefit when combining the SIPS-N and gyrification model, leading to a BAC of 73%. Furthermore, sequentially adding the prognostic performance of the gyrification model on top of the baseline SIPS-N model allowed us to stratify CHR patients into high (83%), medium (40–64%), and low (19%) group, regarding their risk to present moderate/severe negative symptoms after 9 months. These three risk groups also exhibited different responder characteristics to potential interventions. In particular, using data from a previous d-serine trial in CHR patients aiming to treat negative symptoms^47^, we showed that the high-risk group would most benefit from such an intervention (NNT = 1), the medium-risk group at an intermediate level (NNT = 2–3), and the low-risk group least (NNT = 6) (Fig. 2C). This suggests that the assessment of gyrification as a potential complement to initial clinical assessments may be valuable for patient stratification, for example, to assign patients to different treatment arms^13^ in clinical trials.

### Limitations

Some limitations of our study merit comment. Remission of negative symptoms in CHR has been defined as a 20% improvement based on pilot study after d-serine treatment^47^. However, remission of negative symptoms has not been conceptualized yet as categorical outcome and bearing in mind the small size in this study, sequential testing findings should be interpreted with caution. Furthermore, although our models were constructed in a comparatively large CHR sample, they need to be validated in an independent dataset, which may also help to arbitrate between using the SIPS-N or the combined model. For some applications the clinical model alone may already be sufficient to achieve a relevant degree of stratification, which can be integrated easily in care routines, but is associated with a larger degree of uncertainty. For other applications, our models may need to be enriched with more clinical and biological predictors to improve prognosis. The field will have to discuss what accuracy is required for clinical implementation. This should be considered in light of down-stream consequences of being assigned to a specific risk group (for example, increasing monitoring rate, may be ethically justifiable with worse performance compared to administration of medication with potentially serious side effects).

## Conclusions

In conclusion, this study provides a multimodal prognostic model for negative symptoms severity and functional impairments in CHR. Using clinical and gyrification data, we deliver a pragmatic strategy to identify those CHR individuals with clinically relevant negative symptom severity and functional impairments, potentially requiring specialized care. Such multimodal and multistep prognostic testing may help to stratify individual risk profiles and optimize personalized interventions in the future.

## Supplementary information

Supplementary material: Multimodal prognosis of negative symptom severity in individuals at increased risk of developing psychosis

## Data Availability

Analysis code will be provided upon reasonable request to the corresponding author.
