# Supplementary material for: Multimodal prognosis of negative symptom severity in individuals at increased risk of developing psychosis
Source: Transl Psychiatry. 2021 May 24;11:312. doi: 10.1038/s41398-021-01409-4 (PMC8144430; doi:10.1038/s41398-021-01409-4)
Supplement: Supplementary file 1 — Supplementary material: Multimodal prognosis of negative symptom severity in individuals at increased risk of developing psychosis [file 41398_2021_1409_MOESM1_ESM.docx]

**Multimodal prognosis of negative symptom severity in individuals at increased risk of developing psychosis**

Supplementary material

**The PRONIA group**

The authors listed here performed the screening, recruitment, rating, examination, and follow-up of the study participants. They were involved in implementing the examination protocols of the study, setting up its IT infrastructure, and organizing the flow and quality control of the data analyzed in this manuscript between the local study sites and the central study database.

**Department of Psychiatry and Psychotherapy, Ludwig-Maximilian-University, Munich, Germany**

Linda Betz, Anne Erkens, Eva Gussmann, Shalaila Haas, Alkomiet Hasan, Claudius Hoff, Ifrah Khanyaree, Aylin Melo, Susanna Muckenhuber-Sternbauer, Yanis Köhler, Ömer Öztürk, Nora Penzel, David Popovic, Adrian Rangnick, Sebastian von Saldern, Rachele Sanfelici, Moritz Spangemacher, Ana Tupac, Maria Fernanda Urquijo-Castro, Johanna Weiske, Antonia Wosgien, and Camilla Krämer

**Department of Psychiatry and Psychotherapy, University of Cologne, Cologne, Germany**

Karsten Blume, Dennis Hedderich, Dominika Julkowski, Nathalie Kaiser, Thorsten Lichtenstein, Ruth Milz, Alexandra Nikolaides, Tanja Pilgram, Mauro Seves, Silke Vent, and Martina Wassen

**Department of Psychiatry (Psychiatric University Hospital, UPK), University of Basel, Switzerland**

Christina Andreou, Laura Egloff, Fabienne Harrisberger, Ulrike Heitz, Claudia Lenz, Letizia Leanza, Amatya Mackintosh, Renata Smieskova, Erich Studerus, Anna Walter, Sonja Widmayer, Daniel J. Hauke, André Schmidt, and Andreea Diaconescu

**Institute of Mental Health & School of Psychology, University of Birmingham, United Kingdom**

Chris Day, Sian Lowri Griffiths, Mariam Iqbal, Mirabel Pelton, Pavan Mallikarjun, Alexandra Stainton, Ashleigh Lin, Paris Alexandros Lalousis, and Katharine Chisholm

**Department of Psychiatry, University of Turku, Finland**

Alexander Denissoff, Anu Ellilä, Tiina From, Markus Heinimaa, Tuula Ilonen, Päivi Jalo, Heikki Laurikainen, Antti Luutonen, Akseli Mäkela, Janina Paju, Henri Pesonen, Reetta-Liina Säilä, Anna Toivonen, Otto Turtonen

**Department of Psychiatry, Psychiatric University Hospital LVR / Heinrich-Heine-University Düsseldorf, University of Düsseldorf**

Sonja Botterweck, Norman Kluthausen, Gerald Antoch, Julian Caspers, and Hans-Jörg Wittsack

**Department of Basic Medical Science, Neuroscience and Sense Organs, University of Bari Aldo Moro**

Giuseppe Blasi, Giulio Pergola, Grazia Caforio, Leonardo Fazio, Tiziana Quarto, Barbara Gelao, Raffaella Romano, Ileana Andriola, Andrea Falsetti, Marina Barone, Roberta Passiatore, and Marina Sangiuliano

**Department of Psychiatry and Psychotherapy, University of Münster**

Marian Surmann, Olga Bienek, and Udo Dannlowski

**General Electric Global Research Inc., USA**

Ana Beatriz Solana, Manuela Abraham, Nicolas Hehn, and Timo Schirmer

**Workgroup of Paolo Brambilla, University of Milan, Italy**

- Department of Neuroscience and Mental Health, Fondazione IRCCS Ca' Granda Ospedale Maggiore Policlinico, University of Milan, Milan, Italy: Carlo Altamura, Marika Belleri, Francesca Bottinelli, Adele Ferro, and Marta Re
- Programma 2000, Niguarda Hospital, Milan: Emiliano Monzani, and Maurizio Sberna
- San Paolo Hospital, Milan: Giampaolo Perna, Maria Nobile, and Alessandra Alciati
- Villa San Benedetto Menni, Albese con Cassano (CO): Armando D’Agostino, and Lorenzo Del Fabro

**Workgroup of Paolo Brambilla at the University of Udine, Italy**

- Department of Medical Area, University of Udine, Udine, Italy: Matteo Balestrieri, Carolina Bonivento, Giuseppe Cabras, and Franco Fabbro
- IRCCS Scientific Institute E. Medea, Polo FVG: Marco Garzitto, and Sara Piccin

**Specific exclusion criteria for recent-onset psychosis and depression patients**

The participants with recent-onset psychosis patients (ROP) met the following criteria: (a) DSM-IV-TR affective or non-affective psychotic episode (life time, (b) criteria for DSM-IV-TR affective or non-affective psychotic episode fulfilled within past 3 months and (c) onset of psychosis within past 24 months. Specific exclusion criteria for ROP were antipsychotic medication longer than 90 days with a daily dose rate at or above minimum dosage in the ‘1^st^ episode psychosis’ range of the DGPPN S3 guideline.^1^ ROD patients were identified by (a) DSM-IV-TR major depressive episode (lifetime), (b) major depressive disorder criteria fulfilled within past three months and (c) duration of first depressive episode no longer than 24 months. Specific recent-onset depression (ROD) exclusion criteria were: (i) more than 1 major depressive episode, (ii) antipsychotic medication for > 30 days (cumulative number of days) at or above minimum dosage of the ‘1^st^ episode psychosis’ range of the DGPPN S3 guidelines and (iii) any intake of antipsychotic medication within the past 3 months before psychopathological baseline assessments at or above minimum dosage of the ‘1^st^ episode psychosis’ range of the DGPPN S3 guidelines.^1^

**Figure S1.** Schematic Analysis Overview


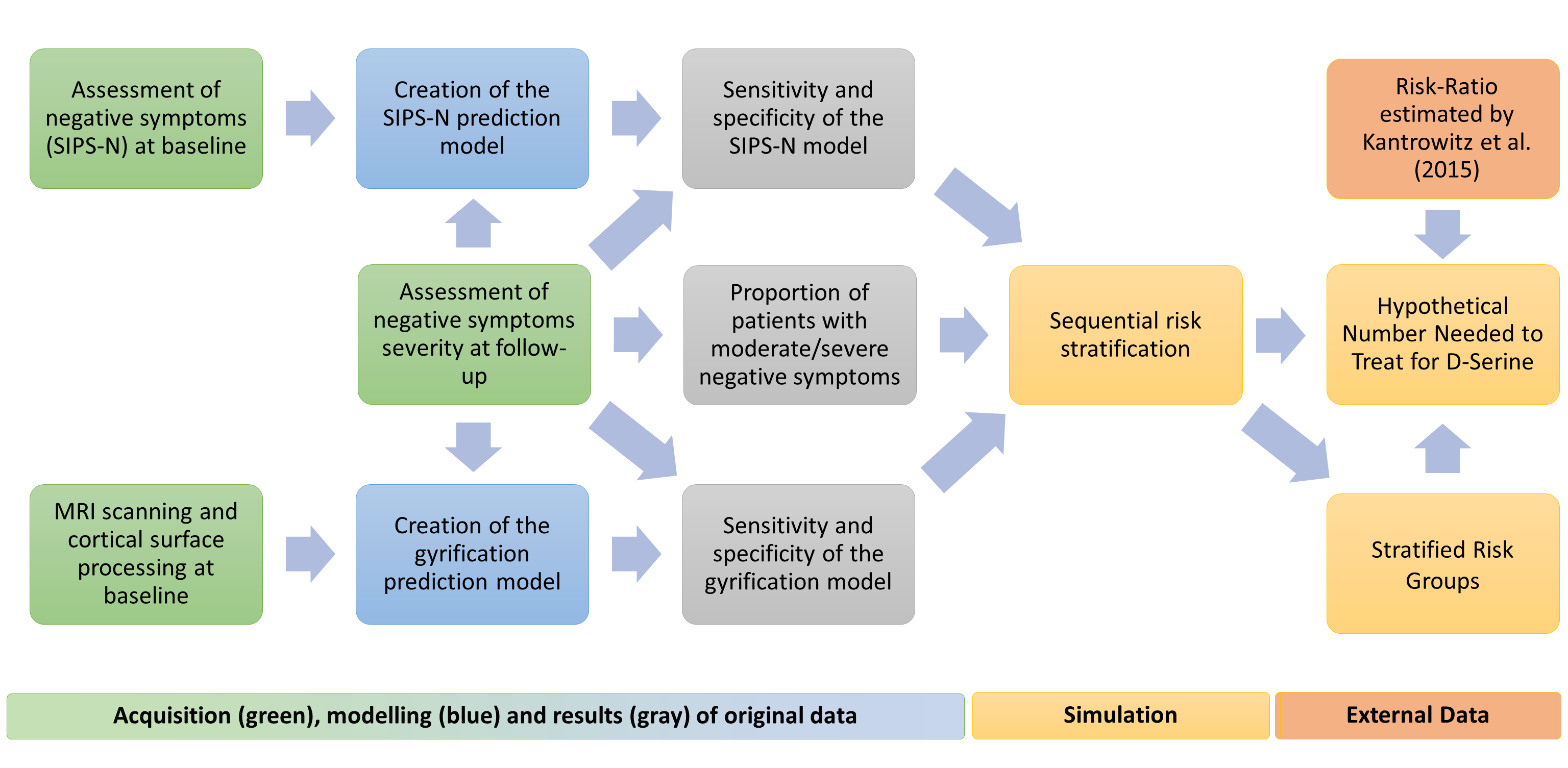


We first used original data (SIPS-N scores and MRI scans at baseline) to create models that predict negative symptom severity at follow-up. Secondly, we calculated the sensitivity and specificity of these models to detect individuals that either would or would not experience moderate/severe negative symptoms after nine month. Finally, we only used these sensitivities and specificities and the proportion of patients with moderate/severe symptoms at follow-up to simulate a sequential risk stratification. This analysis yielded sequentially stratified risk groups and allowed us to estimate a hypothetical number needed to treat for a D-serine intervention based on the risk groups we derived from our data and the risk-ratios of the intervention estimated in a recent clinical pilot trial.^2^ **SIPS-N**: negative symptoms measured the Structured Interview for Psychosis-Risk Syndromes.^3^ **MRI**: magnetic resonance imaging.

**Table S1.** MRI data acquisition.

| **Site** | **Manufacturer** | **Field strength** | **Coil channels** | **Flip angle** | **TR (ms)** | **TE (ms)** | **Voxel size (mm)** | **FOV** | **Slice number** |
| --- | --- | --- | --- | --- | --- | --- | --- | --- | --- |
| Munich | Philips Ingenia | 3T | 32 | 8 | 9.5 | 5.5 | 0.97 x 0.97 x 1.0 | 250 x 250 | 190 |
| Milan | Philips Achieva Intera | 1.5T | 8 | 12 | 8.1 | 3.7 | 0.93 x 0.93 x 1.0 | 240 x 240 | 170 |
| Basel | SIEMENS Verio | 3T | 12 | 8 | 2000 | 3.4 | 1.0 x 1.0 x 1.0 | 256 x 256 | 176 |
| Cologne | Philips Achieva | 3T | 8 | 8 | 9.5 | 5.5 | 0.97 x 0.97 x 1.0 | 250 x 250 | 190 |
| Birmingham | Philips Achieva | 3T | 32 | 8 | 8.4 | 3.8 | 1.0 x 1.0 x 1.0 | 288 x 288 | 175 |
| Turku | Philips Ingenuity | 3T | 32 | 7 | 8.1 | 3.7 | 1.0 x 1.0 x 1.0 | 256 x 256 | 176 |
| Udine | Philips Achieva | 3T | 8 | 12 | 8.1 | 3.7 | 0.93 x 0.93 x 1.0 | 240 x 240 | 170 |

**Table S2.** Participants per study center.

|  | **CHR** | | | **ROP** | | | **ROD** | | |
| --- | --- | --- | --- | --- | --- | --- | --- | --- | --- |
| **Moderate/Severe negative symptoms at T1** | Yes | No | Statistic | Yes | No | Statistic | Yes | No | Statistic |
| Total number participants  (%)  Per Study centre ^a^  Munich  Basel  Udine  Milan  Birmingham  Cologne  Turku | 38 (40%)  12  5  4  5  1  6  5 | 56 (60%)  17  9  8  1  11  5  5 | χ^2^_6_=11.400  p=0.077 | 57 (59%)  18  11  3  7  4  9  5 | 39 (41%)  10  6  3  3  2  9  6 | χ^2^_6_=2.839  p=0.829 | 31 (32%)  15  4  3  3  3  2  1 | 66 (68%)  17  8  12  0  8  14  7 | χ^2^_6_=14.948**p=0.021** |

**^a^** χ^2^ tests between individuals with moderate / severe (any score ≥ 3) and mild (all scores < 3) negative symptoms at follow-up.

**Gyrification computation**

For preprocessing and analysis-steps, pre-set parameters in accordance with the standard protocol (http://www.neuro.uni-jena.de/cat12/CAT12-Manual.pdf) were used, applying default settings unless indicated otherwise. Local (vertex-wise) gyrification indices (GI) were calculated based on the absolute mean curvature approach.^4^ Extraction of the cortical surface resulted in the construction of a mesh of the central surface,^5^ i.e. the surface between the grey/CSF border and the grey matter/white matter boundary. The local absolute mean curvature of this central surface was then computed by averaging the mean curvature values from each vertex point with 3 mm from a given point. In line with recent studies using CAT12,^6, 7^ we applied 15 mm full-width at half maximum (FWHM) smoothing to the GI maps. Gyrification values were then extracted for 68 regions (34 for each hemisphere) using the Desikan-Killiany atlas^8^ as implemented in CAT12.

**Machine learning analysis details**

Preprocessing was embedded in the cross-validation and consisted for the clinical model of feature scaling [-1, 1], regressing out the effects of the covariates age and gender as reported previously,^9^ as well as a subsequent rescaling [-1, 1] to ensure that predictors were in the same range after covariate correction. We then trained an L2-regularized logistic regression using the liblinear implementation (version: 2.2; <https://github.com/cjlin1/liblinear>) with hyperparameter optimisation of the cost parameter in each inner cross-validation fold over the range of 2.^[-5:4], and hyperplane weighing to adjust for the imbalance in the outcome distributions. The final prediction was generated by aggregating the 25% best models into an ensemble to increase robustness. Models were then retrained on the entire inner fold using the optimal parameter and applied to the held-out test fold. Preprocessing of the gyrification features consisted of median standardization, regressing out the effects of the covariates age and gender, and a subsequent re-standardization. Due to the increased number of features, we trained an L1-regularized L2-loss, linear support vector machine classifier, optimising the C-parameter over the range of 2.^[-4:4], with hyperplane weighing and aggregating the 25% best models into an ensemble. Lastly, the combined model was a stacking model taking the predictions of the clinical and gyrification model as inputs. We rescaled the predictions to [-1, 1] and imputed missing predictions using k-nearest-neighbour imputation with *k* = 7, before subjecting them to an L2-regularized logistic regression, optimising the cost over 2.^[-5:4], with hyperplane weighing (no ensembles were generated in the stacking model).

**Tables S3.** Comprehensive overview over clinical and demographic characteristics.

|  | **Clinical high-risk for psychosis** | | | **Recent-onset psychosis** | | | **Recent-onset depression** | | |
| --- | --- | --- | --- | --- | --- | --- | --- | --- | --- |
| **Moderate/Severe negative symptoms at T1** | **Yes** | **No** | **Statistic** | **Yes** | **No** | **Statistic** | **Yes** | **No** | **Statistic** |
| **Total number participants** (%) | 38 (40) | 56 (60) |  | 57 (59) | 39 (41) |  | 31 (32) | 66(68) |  |
| **Age**  mean (SD)^a^ | 23.75  (4.71) | 24.49  (5.69) | t_92_=-0.689  p=0.492 | 24.79  (4.94) | 26.10  (6.08) | t_94_=-1.088  p=0.280 | 26.69  (6.48) | 26.76  (6.00) | t_95_=-0.055  p=0.956 |
| **Sex**  women / men^b^ | 15/23 | 29/27 | χ^2^_1_=1.378  p=0.240 | 16/41 | 19/20 | χ^2^_1_=4.261  **p=0.039** | 15/16 | 40/26 | χ^2^_6_=1.283  p=0.257 |
| **Edinburgh Handedness Score (writing)**  mean (SD)^a^ | 65.28  (75.42) | 77.88  (59.75) | t_86_=-0.837  p=0.406 | 71.82  (67.88) | 79.12  (56.54) | t_89_=-0.559  p=0.577 | 89.29  (39.34) | 77.42  (63.81) | t_88_=1.079  p=0.284 |
| **Years of education**  mean (SD)^a^ | 13.17  (2.88) | 14.02  (3.18) | t_91_=-1.336  p=0.185 | 13.34  (2.92) | 15.13  (3.46) | t_94_=-2.656  **p=0.010** | 14.65  (3.00) | 15.10  (2.97) | t_94_=-0.696  p=0.489 |
| **BDI T0**  median [25^th^ percentile, 75^th^ percentile]^c^ | 28.00 _n=36_  [19.50, 35.25] | 29.00 _n=53_  [9.00, 32.00] | U=851.000  p=0.389 | 21.50 _n=55_  [10.75, 31.00] | 18.50 _n=35_  [6.00, 25.50] | U=758.500  p=0.143 | 26.00 _n=30_  [19.00, 37.50] | 24.00 _n=62_  [14.00, 34.75] | U=704.000  p=0.060 |
| **BDI T1**  median [25^th^ percentile, 75^th^ percentile]^c^ | 19.00 _n=28_  [15.25, 33.25] | 10.00 _n=45_  [4.00, 17.00] | U=251.000  **p<0.001** | 16.00 _n=50_  [7.00, 21.75] | 4.00 _n=29_  [1.75, 7.50] | U=351.500  **p<0.001** | 16.00 _n=27_  [8.50, 23.25] | 11.00 _n=57_  [3.00, 19.00] | U=519.000  **p=0.016** |
| **BDI T1-T0**  median [25^th^ percentile, 75^th^ percentile]^c^ | -2.50 _n=27_  [-9.50, 2.25] | -10.00 _n=45_  [-19.00, -2.00] | U=373.500  **p=0.009** | -3.00 _n=49_  [-14.50, 4.00] | -11.00 _n=27_  [-18.00, -3.50] | U=450.500  **p=0.022** | -9.00 _n=26_  [-19.75, 1.00] | -13.00 _n=55_  [-23.50, -4.00] | U=577.000  p=0.162 |
| **SIPS negative symptoms T0**  median [25^th^ percentile, 75^th^ percentile] ^c^ | 13.50 _n=38_  [5.75, 6.25] | 7.00 _n=56_  [3.00, 10.00] | U=610.000  **p<0.001** | 12.00 _n=57_  [6.75, 17.00] | 7.50 _n=39_  [3.75, 11.00] | U=750.500  **p=0.007** | 9.50 _n=31_  [6.25, 13.75] | 7.00 _n=65_  [4.00, 11.00] | U=754.000  **p=0.047** |
| **SIPS negative symptoms T1**  median [25^th^ percentile, 75^th^ percentile] ^c^ | 7.50 _n=38_  [6.75, 10.00] | 1.00 _n=56_  [0.00, 3.00] | U=33.500  **p<0.001** | 9.00 _n=57_  [6.00, 14.00] | 0.00 _n=39_  [0.00, 2.25] | U=90.000  **p<0.001** | 8.00 _n=31_  [5.25, 10.00] | 0.50 _n=66_  [0.00, 5.00] | U=86.000  **p<0.001** |
| **SIPS negative symptoms T1-T0**  median [25^th^ percentile, 75^th^ percentile] ^c^ | -3.50 _n=38_  [-9.00, 3.00] | -5.00 _n=56_  [-9.00, -1.00] | U=822.500  p=0.062 | -1.50 _n=57_  [-6.00, 2.25] | -6.00 _n=39_  [-10.00, -0.75] | U=656.500  **p=0.001** | -1.00 _n=31_  [-3.75, 1.00] | -6.00 _n=66_  [-10.00, -2.00] | U=557.000  **p<0.001** |
| **SIPS positive symptoms T0**  median [25^th^ percentile, 75^th^ percentile] ^c^ | 8.00 _n=36_  [4.75, 12.00] | 7.00 _n=56_  [3.00, 11.00] | U=989.000  p=0.463 | 17.00 _n=56_  [13.75, 21.00] | 17.50 _n=39_  [12.00, 21.25] | U=1068.500  p=0.859 | 2.00 _n=31_  [1.00, 4.75] | 1.00 _n=66_  [0.00, 3.25] | U=898.500  p=0.324 |
| **SIPS positive symptoms T1**  median [25^th^ percentile, 75^th^ percentile] ^c^ | 6.50 _n=37_  [2.00, 11.25] | 3.00 _n=56_  [0.00, 6.00] | U=686.500  **p=0.006** | 6.00 _n=55_  [1.00, 12.25] | 0.50 _n=37_  [0.00, 4.5] | U=700.000  **p=0.010** | 2.00 _n=31_  [0.00, 3.00] | 0.00 _n=66_  [0.00, 2.00] | U=743.000  **p=0.022** |
| **SIPS positive symptoms T1-T0**  median [25^th^ percentile, 75^th^ percentile] ^c^ | -1.50 _n=37_  [-6.00, 1.25] | -3.00 _n=56_  [-6.00, 0.00] | U=869.000  p=0.188 | -10.00 _n=54_  [-15.25, -6.00] | -12.00 _n=37_  [-18.25, -6.00] | U=770.500  p=0.064 | 0.00 _n=31_  [-2.75, 1.00] | 0.00 _n=66_  [-2.00, 0.00] | U=852.500  p=0.172 |

|  | **Clinical high-risk for psychosis** | | | **Recent-onset psychosis** | | | **Recent-onset depression** | | |
| --- | --- | --- | --- | --- | --- | --- | --- | --- | --- |
| **Moderate/Severe negative symptoms at T1** | **Yes** | **No** | **Statistic** | **Yes** | **No** | **Statistic** | **Yes** | **No** | **Statistic** |
| **SIPS disorganization symptoms T0**  median [25^th^ percentile, 75^th^ percentile] ^c^ | 3.00 _n=38_  [2.00, 6.00] | 2.00 _n=56_  [2.00, 4.00] | U=869.500  p=0.127 | 6.50 _n=57_  [4.00, 10.00] | 3.50 _n=39_  [2.00, 6.25] | U=776.000  **p=0.012** | 2.00 _n=31_  [0.00, 3.00] | 2.00 _n=66_  [0.00, 3.00] | U=978.500  p=0.0816 |
| **SIPS disorganization symptoms T1**  median [25^th^ percentile, 75^th^ percentile] ^c^ | 2.00 _n=38_  [0.00, 3.50] | 0.00 _n=56_  [0.00, 2.00] | U=594.500  **p<0.001** | 3.00 _n=57_  [0.75, 4.00] | 0.00 _n=39_  [0.00, 2.00] | U=508.500  **p<0.001** | 1.00 _n=31_  [0.00, 2.00] | 0.00 _n=66_  [0.00, 1.00] | U=652.500  **p=0.001** |
| **SIPS disorganization symptoms T1-T0** median [25^th^ percentile, 75^th^ percentile] ^c^ | -0.50 _n=38_  [-3.00, 0.00] | -2.00 _n=56_  [-3.00, 0.00] | U=867.000  p=0.122 | -3.00 _n=57_  [-5.00, 0.00] | -3.00 _n=39_  [-5.50, 0.00] | U=986.000  p=0.347 | -1.00 _n=31_  [-2.00, 0.00] | -2.00 _n=66_  [-3.00, 0.00] | U=809.500  p=0.093 |
| **SIPS general symptoms T0**  median [25^th^ percentile, 75^th^ percentile] ^c^ | 8.00 _n=38_  [4.00, 10.25] | 7.00 _n=56_  [5.00, 10.00] | U=912.500  p=0.241 | 7.00 _n=57_  [4.75, 11.00] | 8.00 _n=39_  [4.75, 11.00] | U= 1041.000  p=0.598 | 8.50 _n=31_  [5.25, 10.75] | 7.00 _n=65_  [4.75, 10.25] | U=814.000  p=0.128 |
| **SIPS general symptoms T1**  median [25^th^ percentile, 75^th^ percentile] ^c^ | 5.00 _n=38_  [2.75, 7.25] | 2.00 _n=56_  [0.00, 4.00] | U=396.000  **p<0.001** | 4.50 _n=57_  [2.00, 8.00] | 0.00 _n=39_  [0.00, 2.25] | U=531.000  **p<0.001** | 6.00 _n=31_  [2.25, 7.75] | 1.00 _n=66_  [0.00, 4.00] | U=417.500  **p<0.001** |
| **SIPS general symptoms T1-T0**  median [25^th^ percentile, 75^th^ percentile] ^c^ | -1.50 _n=38_  [-4.00, 0.00] | -5.00 _n=56_  [-9.00, -1.00] | U=684.500  **p=0.003** | -2.00 _n=57_  [-5.00, 0.00] | -5.50 _n=39_  [-9.50, -3.00] | U=738.500  **p=0.005** | -2.00 _n=31_  [-5.75, 1.00] | -4.50 _n=66_  [-9.00, -1.75] | U=686.000  **p=0.009** |
| **Global functioning: Social T0**  median [25^th^ percentile, 75^th^ percentile] ^c^ | 6.00 _n=38_  [5.00, 7.00] | 7.00 _n=56_  [6.00, 8.00] | U=507.500  **p<0.001** | 5.50 _n=57_  [5.00, 7.00] | 6.00 _n=39_  [5.00, 7.00] | U=779.000  **p=0.011** | 6.00 _n=31_  [5.00, 7.00] | 7.00 _n=65_  [6.00, 8.00] | U=719.000  **p=0.020** |
| **Global functioning: Social T1**  median [25^th^ percentile, 75^th^ percentile] ^c^ | 6.00 _n=37_  [6.00, 7.00] | 8.00 _n=56_  [7.00, 8.00] | U=356.000  **p<0.001** | 7.00 _n=56_  [5.00, 8.00] | 8.00 _n=39_  [7.00, 8.00] | U=668.000  **p<0.001** | 6.50 _n=31_  [5.25, 8.00] | 8.00 _n=66_  [7.00, 8.00] | U=557.500  **p<0.001** |
| **Global functioning: Social T1 –T0**  median [25^th^ percentile, 75^th^ percentile] ^c^ | 0.00 _n=37_  [0.00, 1.00] | 1.00 _n=56_  [0.00, 1.00] | U=987.500  p=0.688 | 1.00 _n=56_  [0.00, 1.00] | 1.00 _n=39_  [0.75, 2.00] | U=889.500  p=0.113 | 0.00 _n=31_  [0.00, 1.00] | 1.00 _n=65_  [0.00, 2.00] | U=793.500  p=0.079 |
| **Global functioning: Role T0**  median [25^th^ percentile, 75^th^ percentile] ^c^ | 5.00 _n=38_  [5.00, 6.00] | 7.00 _n=56_  [6.00, 8.00] | U=498.000  **p<0.001** | 5.00 _n=57_  [4.00, 6.00] | 6.00 _n=39_  [5.00, 7.00] | U=706.500  **p=0.002** | 6.50 _n=31_  [5.25, 8.00] | 7.00 _n=65_  [6.00, 7.00] | U=948.500  p=0.637 |
| **Global functioning: Role T1**  median [25^th^ percentile, 75^th^ percentile] ^c^ | 6.00 _n=37_  [4.00, 7.00] | 8.00 _n=56_  [7.00, 8.00] | U=278.500  **P<0.001** | 6.00 _n=56_  [4.00, 7.00] | 7.50 _n=39_  [6.00, 8.00] | U=605.000  **p<0.001** | 7.00 _n=31_  [6.00, 8.00] | 8.00 _n=66_  [7.00, 8.00] | U=531.000  **p<0.001** |
| **Global functioning: Role T1- T0**  median [25^th^ percentile, 75^th^ percentile] ^c^ | 0.00 _n=37_  [-1.00, 1.50] | 1.00 _n=56_  [0.00, 1.75] | U=780.500  **p=0.0038** | 0.50 _n=56_  [0.00, 1.00] | 1.00 _n=39_  [0.00, 2.25] | U=916.500  p=0.171 | -0.50 _n=31_  [-1.00, 1.00] | 1.00 _n=65_  [0.00, 2.00] | U=650.500  **p=0.004** |

**BDI**: Becks depression inventory.^10^ **SIPS**: structured interview for prodromal syndromes.^3^ **T0**: baseline assessment. **T1**: 9-month follow-up assessment. ^a^ two-sample t-tests between individuals with moderate / severe (any score ≥ 3) and mild (all scores < 3) negative symptoms at follow-up. ^b^ χ^2^ tests between individuals with moderate / severe (any score ≥ 3) and mild (all scores < 3) negative symptoms at follow-up. ^c^ two-sample Mann-Whitney-U-tests between individuals with moderate / severe (any score ≥ 3) and mild (all scores < 3) negative symptoms at follow-up.

**Figure S2.** Clinical scores at baseline and follow-up in clinical high-risk individuals.


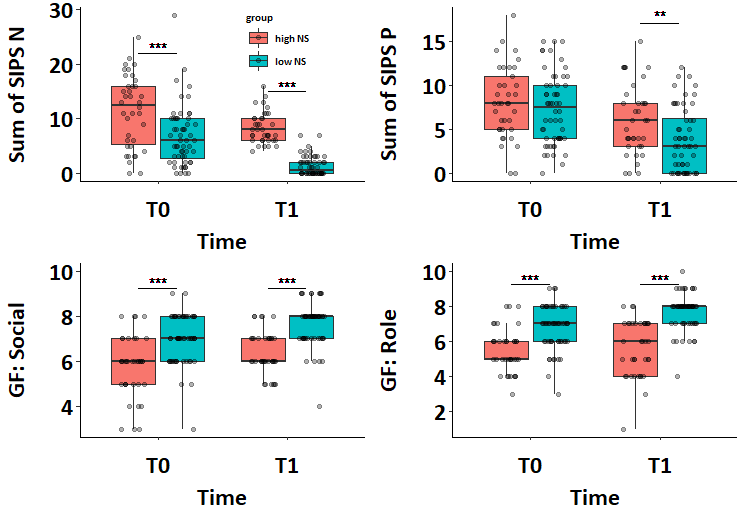


**High NS**: Clinical high-risk individuals with at least moderate to severe negative symptoms (NS) according to the structured interview for prodromal syndromes (SIPS)^3^, specifically, any score ≥ 3 at follow-up assessment. **Low NS**: Clinical high-risk individuals with mild negative symptoms (all scores < 3) at follow-up. **Sum of** **SIPS N**: Sum of SIPS negative symptom items (N1, N2; N3, N4 and N6). **Sum of** **SIPS P**: Sum of SIPS positive symptom items. **GF: Social**: Global Functioning: Social scale.^11^ **GF: Role**: Global Functioning: Role scale.^11^ **T0**: Baseline assessment. **T1**: 9-month follow-up assessment. Significant at (*****) *p* < 0**.**05, (******), and *p* < 0**.**01, (***) *p* < 0**.**001 using two-sample Mann-Whitney-U-test.

**Figure S3.** Generalization performances.


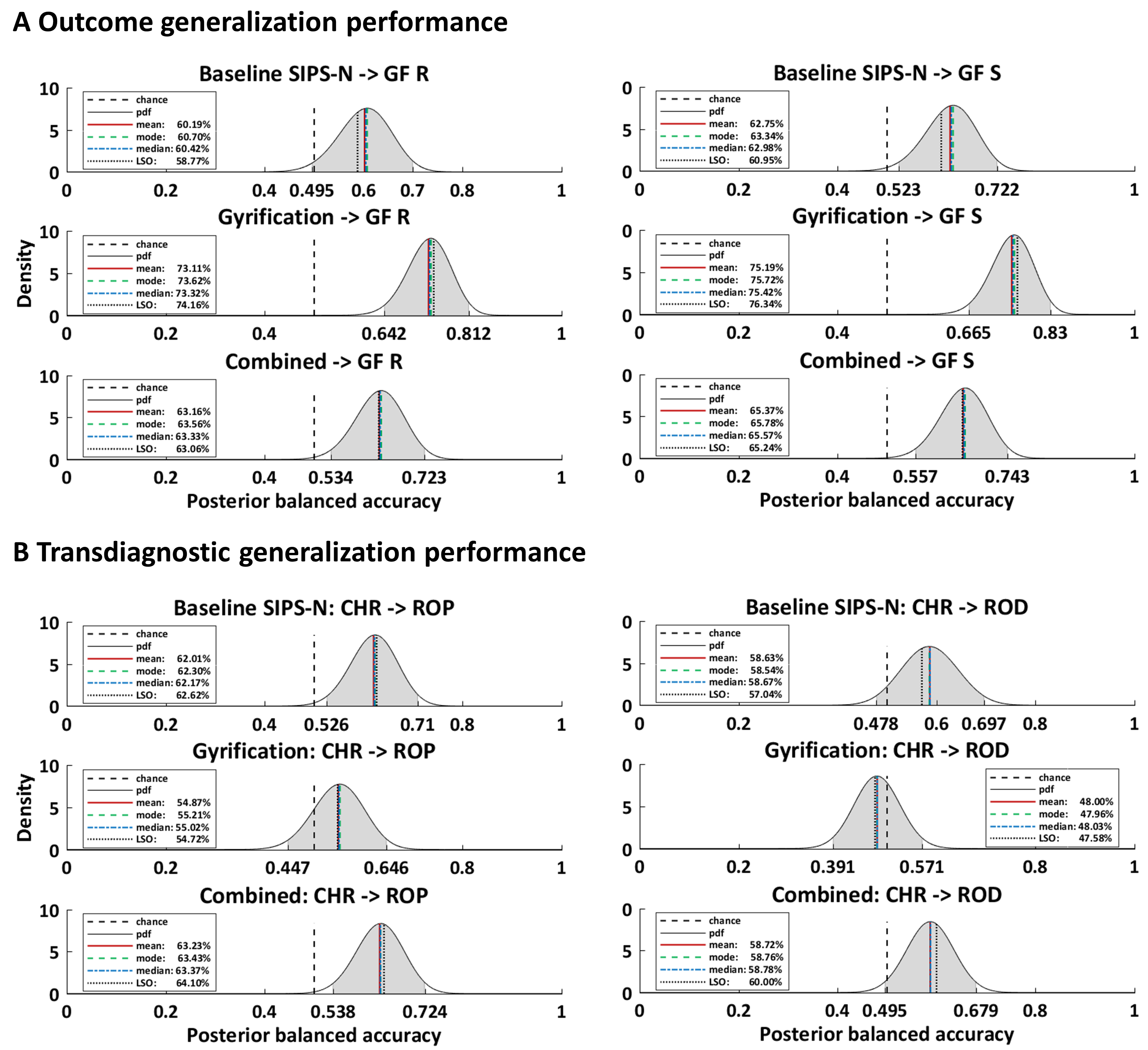


**A** Generalization performance, when employing negative symptom models to predict role (left) and social functioning (right). Posterior balanced accuracy distributions of baseline negative symptom (upper panel), gyrification (middle panel), and combined model (lower panel). Shaded gray area indicates 95% of the probability mass of the respective posterior distribution over the balanced accuracy. **B** Transdiagnostic generalization performance to recent-onset psychosis (left) and depression (right). **pdf**: probability density function. **LSO**: leave-site-out cross validation point estimate. **GF R**: global functioning role subscale.^11^ **GF S:** global functioning social subscale.^11^ **CHR**: clinical high-risk. **ROP**: recent-onset psychosis. **ROP**: recent-onset depression. **SIPS-N**: negative symptoms measured the Structured Interview for Psychosis-Risk Syndromes.^3^

**Table S4.** Gyrification scores in each group.

|  | **Clinical high-risk psychosis** | | | **Recent-onset psychosis** | | | **Recent-onset depression** | | |
| --- | --- | --- | --- | --- | --- | --- | --- | --- | --- |
| **Moderate/Severe**  **negative symptoms at T1** | **Yes** | **No** | **Statistic** | **Yes** | **No** | **Statistic** | **Yes** | **No** | **Statistic** |
| lbankssts mean(SD)^a^ | 0.0161 (0.0020) | 0.0171 (0.0024) | t_92_=-2.205 **p=0.030** | 0.0167 (0.0018) | 0.0169 (0.0020) | t_94_=-0.570 p=0.570 | 0.0167 (0.0021) | 0.0167 (0.0020) | t_95_=0.082 p=0.935 |
| rbankssts mean(SD)^a^ | 0.0160 (0.0021) | 0.0169 (0.0023) | t_92_=-1.925 p=0.057 | 0.0163 (0.0019) | 0.0168 (0.0016) | t_94_=-1.459 p=0.148 | 0.0166 (0.0018) | 0.0166 (0.0019) | t_95_=-0.073 p=0.942 |
| lcaudalanteriorcingulate mean(SD)^a^ | 0.0180 (0.0028) | 0.0189 (0.0029) | t_92_=-1.506 p=0.135 | 0.0179 (0.0025) | 0.0183 (0.0024) | t_94_=-0.831 p=0.408 | 0.0187 (0.0023) | 0.0187 (0.0026) | t_95_=-0.055 p=0.957 |
| rcaudalanteriorcingulate mean(SD)^a^ | 0.0183 (0.0026) | 0.0190 (0.0029) | t_92_=-1.238 p=0.219 | 0.0183 (0.0019) | 0.0187 (0.0021) | t_94_=-1.050 p=0.297 | 0.0185 (0.0020) | 0.0189 (0.0025) | t_95_=-0.832 p=0.407 |
| lcaudalmiddlefrontal mean(SD)^a^ | 0.0175 (0.0024) | 0.0174 (0.0022) | t_92_=0.235 p=0.815 | 0.0173 (0.0017) | 0.0175 (0.0020) | t_94_=-0.557 p=0.579 | 0.0177 (0.0020) | 0.0179 (0.0022) | t_95_=-0.462 p=0.645 |
| rcaudalmiddlefrontal mean(SD)^a^ | 0.0171 (0.0023) | 0.0175 (0.0023) | t_92_=-0.647 p=0.519 | 0.0169 (0.0016) | 0.0173 (0.0018) | t_94_=-1.228 p=0.222 | 0.0173 (0.0018) | 0.0175 (0.0021) | t_95_=-0.388 p=0.699 |
| lcuneus mean(SD)^a^ | 0.0193 (0.0024) | 0.0200 (0.0033) | t_92_=-1.163 p=0.248 | 0.0190 (0.0018) | 0.0198 (0.0022) | t_94_=-1.871 p=0.064 | 0.0194 (0.0019) | 0.0201 (0.0023) | t_95_=-1.422 p=0.158 |
| rcuneus mean(SD)^a^ | 0.0195 (0.0026) | 0.0198 (0.0025) | t_92_=-0.684 p=0.495 | 0.0195 (0.0019) | 0.0196 (0.0022) | t_94_=-0.397 p=0.692 | 0.0197 (0.0018) | 0.0200 (0.0022) | t_95_=-0.759 p=0.449 |
| lentorhinal mean(SD)^a^ | 0.0154 (0.0019) | 0.0159 (0.0024) | t_92_=-1.190 p=0.237 | 0.0156 (0.0022) | 0.0155 (0.0021) | t_94_=0.088 p=0.930 | 0.0153 (0.0015) | 0.0161 (0.0020) | t_95_=-1.962 p=0.053 |
| rentorhinal mean(SD)^a^ | 0.0156 (0.0021) | 0.0162 (0.0026) | t_92_=-1.165 p=0.247 | 0.0156 (0.0019) | 0.0160 (0.0021) | t_94_=-0.897 p=0.372 | 0.0160 (0.0018) | 0.0161 (0.0022) | t_95_=-0.235 p=0.814 |
| lfusiform mean(SD)^a^ | 0.0172 (0.0021) | 0.0180 (0.0027) | t_92_=-1.533 p=0.129 | 0.0173 (0.0016) | 0.0176 (0.0019) | t_94_=-0.728 p=0.468 | 0.0176 (0.0018) | 0.0179 (0.0022) | t_95_=-0.677 p=0.500 |
| rfusiform mean(SD)^a^ | 0.0167 (0.0022) | 0.0174 (0.0024) | t_92_=-1.390 p=0.168 | 0.0168 (0.0019) | 0.0171 (0.0020) | t_94_=-0.699 p=0.486 | 0.0171 (0.0018) | 0.0177 (0.0022) | t_95_=-1.360 p=0.177 |
| linferiorparietal mean(SD)^a^ | 0.0179 (0.0022) | 0.0185 (0.0024) | t_92_=-1.264 p=0.210 | 0.0179 (0.0017) | 0.0181 (0.0019) | t_94_=-0.533 p=0.595 | 0.0184 (0.0019) | 0.0185 (0.0021) | t_95_=-0.361 p=0.719 |
| rinferiorparietal mean(SD)^a^ | 0.0179 (0.0022) | 0.0184 (0.0026) | t_92_=-0.903 p=0.369 | 0.0178 (0.0018) | 0.0182 (0.0018) | t_94_=-0.877 p=0.382 | 0.0181 (0.0017) | 0.0184 (0.0022) | t_95_=-0.665 p=0.508 |
|  | **Clinical high-risk psychosis** | | | **Recent-onset psychosis** | | | **Recent-onset depression** | | |
| **Moderate/Severe**  **negative symptoms at T1** | **Yes** | **No** | **Statistic** | **Yes** | **No** | **Statistic** | **Yes** | **No** | **Statistic** |
| linferiortemporal mean(SD)^a^ | 0.0176 (0.0022) | 0.0182 (0.0028) | t_92_=-1.033 p=0.304 | 0.0175 (0.0018) | 0.0179 (0.0018) | t_94_=-0.972 p=0.334 | 0.0178 (0.0017) | 0.0181 (0.0019) | t_95_=-0.559 p=0.577 |
| rinferiortemporal mean(SD)^a^ | 0.0176 (0.0024) | 0.0181 (0.0023) | t_92_=-0.950 p=0.344 | 0.0176 (0.0016) | 0.0179 (0.0017) | t_94_=-0.887 p=0.377 | 0.0179 (0.0017) | 0.0182 (0.0022) | t_95_=-0.602 p=0.549 |
| listhmuscingulate mean(SD)^a^ | 0.0182 (0.0023) | 0.0184 (0.0024) | t_92_=-0.492 p=0.624 | 0.0181 (0.0020) | 0.0186 (0.0020) | t_94_=-1.050 p=0.297 | 0.0184 (0.0016) | 0.0188 (0.0024) | t_95_=-0.866 p=0.389 |
| risthmuscingulate mean(SD)^a^ | 0.0184 (0.0021) | 0.0189 (0.0028) | t_92_=-0.861 p=0.392 | 0.0182 (0.0018) | 0.0190 (0.0021) | t_94_=-1.974 p=0.051 | 0.0191 (0.0021) | 0.0190 (0.0023) | t_95_=0.242 p=0.809 |
| llateraloccipital mean(SD)^a^ | 0.0194 (0.0025) | 0.0199 (0.0026) | t_92_=-0.840 p=0.403 | 0.0192 (0.0017) | 0.0195 (0.0019) | t_94_=-0.881 p=0.380 | 0.0195 (0.0018) | 0.0199 (0.0022) | t_95_=-0.897 p=0.372 |
| rlateraloccipital mean(SD)^a^ | 0.0194 (0.0025) | 0.0199 (0.0026) | t_92_=-0.948 p=0.345 | 0.0193 (0.0018) | 0.0197 (0.0019) | t_94_=-0.998 p=0.321 | 0.0196 (0.0017) | 0.0200 (0.0022) | t_95_=-0.754 p=0.453 |
| llateralorbitofrontal mean(SD)^a^ | 0.0184 (0.0023) | 0.0187 (0.0020) | t_92_=-0.814 p=0.418 | 0.0183 (0.0019) | 0.0186 (0.0017) | t_94_=-0.861 p=0.392 | 0.0186 (0.0017) | 0.0189 (0.0021) | t_95_=-0.730 p=0.467 |
| rlateralorbitofrontal mean(SD)^a^ | 0.0188 (0.0024) | 0.0192 (0.0023) | t_92_=-0.841 p=0.403 | 0.0186 (0.0019) | 0.0188 (0.0019) | t_94_=-0.679 p=0.499 | 0.0192 (0.0020) | 0.0192 (0.0021) | t_95_=-0.064 p=0.949 |
| llingual mean(SD)^a^ | 0.0183 (0.0023) | 0.0184 (0.0024) | t_92_=-0.222 p=0.825 | 0.0181 (0.0018) | 0.0181 (0.0018) | t_94_=-0.163 p=0.871 | 0.0184 (0.0018) | 0.0187 (0.0022) | t_95_=-0.845 p=0.400 |
| rlingual mean(SD)^a^ | 0.0183 (0.0022) | 0.0185 (0.0027) | t_92_=-0.369 p=0.713 | 0.0183 (0.0018) | 0.0184 (0.0019) | t_94_=-0.333 p=0.740 | 0.0184 (0.0020) | 0.0190 (0.0022) | t_95_=-1.127 p=0.262 |
| lmedialorbitofrontal mean(SD)^a^ | 0.0179 (0.0023) | 0.0183 (0.0024) | t_92_=-0.871 p=0.386 | 0.0178 (0.0019) | 0.0182 (0.0019) | t_94_=-1.011 p=0.315 | 0.0181 (0.0016) | 0.0186 (0.0023) | t_95_=-1.066 p=0.289 |
| rmedialorbitofrontal mean(SD)^a^ | 0.0183 (0.0022) | 0.0188 (0.0025) | t_92_=-0.945 p=0.347 | 0.0181 (0.0017) | 0.0184 (0.0018) | t_94_=-0.748 p=0.456 | 0.0187 (0.0018) | 0.0188 (0.0021) | t_95_=-0.292 p=0.771 |
| lmiddletemporal mean(SD)^a^ | 0.0171 (0.0021) | 0.0178 (0.0029) | t_92_=-1.244 p=0.216 | 0.0172 (0.0017) | 0.0174 (0.0015) | t_94_=-0.531 p=0.597 | 0.0176 (0.0018) | 0.0178 (0.0018) | t_95_=-0.483 p=0.630 |
| rmiddletemporal mean(SD)^a^ | 0.0169 (0.0020) | 0.0173 (0.0023) | t_92_=-0.974 p=0.333 | 0.0168 (0.0014) | 0.0170 (0.0015) | t_94_=-0.677 p=0.500 | 0.0170 (0.0015) | 0.0172 (0.0019) | t_95_=-0.550 p=0.584 |
| lparahippocampal mean(SD)^a^ | 0.0148 (0.0021) | 0.0156 (0.0030) | t_92_=-1.504 p=0.136 | 0.0152 (0.0023) | 0.0157 (0.0024) | t_94_=-1.121 p=0.265 | 0.0153 (0.0020) | 0.0156 (0.0026) | t_95_=-0.601 p=0.549 |
|  | **Clinical high-risk psychosis** | | | **Recent-onset psychosis** | | | **Recent-onset depression** | | |
| **Moderate/Severe**  **negative symptoms at T1** | **Yes** | **No** | **Statistic** | **Yes** | **No** | **Statistic** | **Yes** | **No** | **Statistic** |
| rparahippocampal mean(SD)^a^ | 0.0153 (0.0020) | 0.0160 (0.0028) | t_92_=-1.231 p=0.221 | 0.0156 (0.0019) | 0.0161 (0.0022) | t_94_=-1.208 p=0.230 | 0.0159 (0.0016) | 0.0162 (0.0024) | t_95_=-0.491 p=0.625 |
| lparacentral mean(SD)^a^ | 0.0164 (0.0025) | 0.0168 (0.0026) | t_92_=-0.768 p=0.445 | 0.0164 (0.0019) | 0.0165 (0.0017) | t_94_=-0.271 p=0.787 | 0.0163 (0.0019) | 0.0166 (0.0022) | t_95_=-0.707 p=0.481 |
| rparacentral mean(SD)^a^ | 0.0172 (0.0024) | 0.0175 (0.0022) | t_92_=-0.615 p=0.540 | 0.0169 (0.0016) | 0.0170 (0.0017) | t_94_=-0.379 p=0.706 | 0.0172 (0.0019) | 0.0175 (0.0021) | t_95_=-0.491 p=0.624 |
| lparsopercularis mean(SD)^a^ | 0.0169 (0.0022) | 0.0171 (0.0021) | t_92_=-0.552 p=0.582 | 0.0168 (0.0018) | 0.0173 (0.0017) | t_94_=-1.112 p=0.269 | 0.0172 (0.0018) | 0.0174 (0.0021) | t_95_=-0.574 p=0.568 |
| rparsopercularis mean(SD)^a^ | 0.0174 (0.0023) | 0.0177 (0.0023) | t_92_=-0.580 p=0.563 | 0.0169 (0.0017) | 0.0173 (0.0019) | t_94_=-0.963 p=0.338 | 0.0174 (0.0019) | 0.0177 (0.0020) | t_95_=-0.670 p=0.505 |
| lparsorbitalis mean(SD)^a^ | 0.0183 (0.0025) | 0.0182 (0.0022) | t_92_=0.217 p=0.828 | 0.0182 (0.0020) | 0.0186 (0.0021) | t_94_=-0.911 p=0.365 | 0.0183 (0.0014) | 0.0187 (0.0021) | t_95_=-1.031 p=0.305 |
| rparsorbitalis mean(SD)^a^ | 0.0184 (0.0023) | 0.0188 (0.0026) | t_92_=-0.739 p=0.462 | 0.0183 (0.0021) | 0.0185 (0.0021) | t_94_=-0.499 p=0.619 | 0.0184 (0.0019) | 0.0188 (0.0020) | t_95_=-0.824 p=0.412 |
| lparstriangularis mean(SD)^a^ | 0.0184 (0.0024) | 0.0181 (0.0022) | t_92_=0.729 p=0.468 | 0.0179 (0.0019) | 0.0182 (0.0020) | t_94_=-0.843 p=0.401 | 0.0185 (0.0022) | 0.0188 (0.0023) | t_95_=-0.510 p=0.611 |
| rparstriangularis mean(SD)^a^ | 0.0179 (0.0023) | 0.0183 (0.0023) | t_92_=-0.743 p=0.459 | 0.0178 (0.0018) | 0.0181 (0.0020) | t_94_=-0.773 p=0.441 | 0.0180 (0.0017) | 0.0183 (0.0022) | t_95_=-0.601 p=0.549 |
| lpericalcarine mean(SD)^a^ | 0.0191 (0.0022) | 0.0200 (0.0026) | t_92_=-1.706 p=0.091 | 0.0195 (0.0020) | 0.0198 (0.0019) | t_94_=-0.880 p=0.381 | 0.0194 (0.0018) | 0.0200 (0.0026) | t_95_=-1.044 p=0.299 |
| rpericalcarine mean(SD)^a^ | 0.0191 (0.0022) | 0.0196 (0.0025) | t_92_=-1.034 p=0.304 | 0.0191 (0.0019) | 0.0193 (0.0021) | t_94_=-0.542 p=0.589 | 0.0194 (0.0019) | 0.0197 (0.0024) | t_95_=-0.662 p=0.509 |
| lpostcentral mean(SD)^a^ | 0.0167 (0.0020) | 0.0170 (0.0024) | t_92_=-0.763 p=0.447 | 0.0165 (0.0015) | 0.0169 (0.0017) | t_94_=-1.319 p=0.190 | 0.0166 (0.0017) | 0.0171 (0.0019) | t_95_=-1.283 p=0.202 |
| rpostcentral mean(SD)^a^ | 0.0167 (0.0021) | 0.0168 (0.0024) | t_92_=-0.286 p=0.775 | 0.0162 (0.0016) | 0.0166 (0.0016) | t_94_=-1.254 p=0.213 | 0.0165 (0.0016) | 0.0168 (0.0019) | t_95_=-0.607 p=0.545 |
| lposteriorcingulate mean(SD)^a^ | 0.0179 (0.0023) | 0.0186 (0.0025) | t_92_=-1.334 p=0.185 | 0.0180 (0.0021) | 0.0183 (0.0017) | t_94_=-0.920 p=0.360 | 0.0183 (0.0021) | 0.0186 (0.0022) | t_95_=-0.551 p=0.583 |
| rposteriorcingulate mean(SD)^a^ | 0.0182 (0.0021) | 0.0185 (0.0026) | t_92_=-0.645 p=0.521 | 0.0181 (0.0019) | 0.0184 (0.0019) | t_94_=-0.694 p=0.489 | 0.0183 (0.0016) | 0.0187 (0.0023) | t_95_=-0.909 p=0.366 |
|  | **Clinical high-risk psychosis** | | | **Recent-onset psychosis** | | | **Recent-onset depression** | | |
| **Moderate/Severe**  **negative symptoms at T1** | **Yes** | **No** | **Statistic** | **Yes** | **No** | **Statistic** | **Yes** | **No** | **Statistic** |
| lprecentral mean(SD)^a^ | 0.0168 (0.0021) | 0.0171 (0.0020) | t_92_=-0.700 p=0.486 | 0.0166 (0.0017) | 0.0168 (0.0016) | t_94_=-0.361 p=0.719 | 0.0168 (0.0015) | 0.0171 (0.0019) | t_95_=-0.733 p=0.465 |
| rprecentral mean(SD)^a^ | 0.0168 (0.0022) | 0.0173 (0.0023) | t_92_=-1.082 p=0.282 | 0.0166 (0.0016) | 0.0168 (0.0017) | t_94_=-0.451 p=0.653 | 0.0169 (0.0015) | 0.0172 (0.0020) | t_95_=-0.704 p=0.483 |
| lprecuneus mean(SD)^a^ | 0.0185 (0.0024) | 0.0188 (0.0028) | t_92_=-0.596 p=0.552 | 0.0184 (0.0018) | 0.0186 (0.0019) | t_94_=-0.548 p=0.585 | 0.0187 (0.0019) | 0.0189 (0.0023) | t_95_=-0.457 p=0.649 |
| rprecuneus mean(SD)^a^ | 0.0185 (0.0022) | 0.0188 (0.0024) | t_92_=-0.595 p=0.553 | 0.0183 (0.0018) | 0.0186 (0.0019) | t_94_=-0.668 p=0.506 | 0.0186 (0.0016) | 0.0189 (0.0021) | t_95_=-0.623 p=0.535 |
| lrostralanteriorcingulate mean(SD)^a^ | 0.0191 (0.0022) | 0.0198 (0.0029) | t_92_=-1.207 p=0.231 | 0.0191 (0.0022) | 0.0194 (0.0023) | t_94_=-0.752 p=0.454 | 0.0192 (0.0020) | 0.0199 (0.0024) | t_95_=-1.430 p=0.156 |
| rrostralanteriorcingulate mean(SD)^a^ | 0.0192 (0.0027) | 0.0194 (0.0028) | t_92_=-0.496 p=0.621 | 0.0190 (0.0023) | 0.0191 (0.0019) | t_94_=-0.192 p=0.848 | 0.0193 (0.0021) | 0.0196 (0.0027) | t_95_=-0.639 p=0.524 |
| lrostralmiddlefrontal mean(SD)^a^ | 0.0192 (0.0023) | 0.0193 (0.0022) | t_92_=-0.402 p=0.689 | 0.0190 (0.0019) | 0.0192 (0.0020) | t_94_=-0.683 p=0.496 | 0.0193 (0.0018) | 0.0196 (0.0022) | t_95_=-0.707 p=0.481 |
| rrostralmiddlefrontal mean(SD)^a^ | 0.0189 (0.0023) | 0.0194 (0.0025) | t_92_=-0.972 p=0.334 | 0.0188 (0.0019) | 0.0192 (0.0020) | t_94_=-0.952 p=0.344 | 0.0193 (0.0019) | 0.0195 (0.0021) | t_95_=-0.504 p=0.615 |
| lsuperiorfrontal mean(SD)^a^ | 0.0175 (0.0022) | 0.0178 (0.0023) | t_92_=-0.670 p=0.505 | 0.0174 (0.0017) | 0.0176 (0.0018) | t_94_=-0.602 p=0.548 | 0.0177 (0.0017) | 0.0179 (0.0020) | t_95_=-0.499 p=0.619 |
| rsuperiorfrontal mean(SD)^a^ | 0.0178 (0.0021) | 0.0181 (0.0024) | t_92_=-0.723 p=0.471 | 0.0175 (0.0017) | 0.0177 (0.0018) | t_94_=-0.588 p=0.558 | 0.0180 (0.0017) | 0.0181 (0.0020) | t_95_=-0.222 p=0.825 |
| lsuperiorparietal mean(SD)^a^ | 0.0184 (0.0022) | 0.0190 (0.0030) | t_92_=-1.150 p=0.253 | 0.0184 (0.0019) | 0.0187 (0.0020) | t_94_=-0.635 p=0.527 | 0.0188 (0.0019) | 0.0189 (0.0021) | t_95_=-0.149 p=0.882 |
| rsuperiorparietal mean(SD)^a^ | 0.0185 (0.0023) | 0.0190 (0.0026) | t_92_=-0.969 p=0.335 | 0.0181 (0.0018) | 0.0186 (0.0020) | t_94_=-1.282 p=0.203 | 0.0187 (0.0019) | 0.0188 (0.0021) | t_95_=-0.316 p=0.753 |
| lsuperiortemporal mean(SD)^a^ | 0.0159 (0.0020) | 0.0164 (0.0022) | t_92_=-1.084 p=0.281 | 0.0157 (0.0015) | 0.0160 (0.0016) | t_94_=-1.027 p=0.307 | 0.0159 (0.0017) | 0.0162 (0.0018) | t_95_=-0.665 p=0.508 |
| rsuperiortemporal mean(SD)^a^ | 0.0158 (0.0021) | 0.0162 (0.0020) | t_92_=-0.948 p=0.345 | 0.0156 (0.0016) | 0.0161 (0.0016) | t_94_=-1.274 p=0.206 | 0.0161 (0.0016) | 0.0162 (0.0018) | t_95_=-0.126 p=0.900 |
| lsupramarginal mean(SD)^a^ | 0.0180 (0.0021) | 0.0185 (0.0022) | t_92_=-1.160 p=0.249 | 0.0179 (0.0018) | 0.0184 (0.0018) | t_94_=-1.258 p=0.211 | 0.0182 (0.0018) | 0.0185 (0.0021) | t_95_=-0.689 p=0.493 |
|  | **Clinical high-risk psychosis** | | | **Recent-onset psychosis** | | | **Recent-onset depression** | | |
| **Moderate/Severe**  **negative symptoms at T1** | **Yes** | **No** | **Statistic** | **Yes** | **No** | **Statistic** | **Yes** | **No** | **Statistic** |
| rsupramarginal mean(SD)^a^ | 0.0179 (0.0022) | 0.0183 (0.0023) | t_92_=-0.818 p=0.415 | 0.0178 (0.0018) | 0.0180 (0.0018) | t_94_=-0.685 p=0.495 | 0.0181 (0.0018) | 0.0184 (0.0022) | t_95_=-0.641 p=0.523 |
| lfrontalpole mean(SD)^a^ | 0.0201 (0.0026) | 0.0206 (0.0028) | t_92_=-0.716 p=0.476 | 0.0194 (0.0020) | 0.0200 (0.0020) | t_94_=-1.434 p=0.155 | 0.0204 (0.0022) | 0.0204 (0.0023) | t_95_=-0.077 p=0.939 |
| rfrontalpole mean(SD)^a^ | 0.0203 (0.0024) | 0.0208 (0.0026) | t_92_=-0.869 p=0.387 | 0.0199 (0.0021) | 0.0210 (0.0024) | t_94_=-2.382 p=0.019 | 0.0205 (0.0020) | 0.0209 (0.0022) | t_95_=-0.783 p=0.435 |
| ltemporalpole mean(SD)^a^ | 0.0156 (0.0022) | 0.0160 (0.0026) | t_92_=-0.859 p=0.392 | 0.0156 (0.0020) | 0.0155 (0.0018) | t_94_=0.286 p=0.776 | 0.0157 (0.0019) | 0.0159 (0.0020) | t_95_=-0.619 p=0.537 |
| rtemporalpole mean(SD)^a^ | 0.0158 (0.0020) | 0.0165 (0.0025) | t_92_=-1.430 p=0.156 | 0.0162 (0.0019) | 0.0162 (0.0019) | t_94_=-0.165 p=0.870 | 0.0160 (0.0018) | 0.0165 (0.0019) | t_95_=-1.211 p=0.229 |
| ltransversetemporal mean(SD)^a^ | 0.0161 (0.0029) | 0.0165 (0.0022) | t_92_=-0.745 p=0.458 | 0.0160 (0.0023) | 0.0155 (0.0020) | t_94_=1.106 p=0.272 | 0.0160 (0.0016) | 0.0167 (0.0024) | t_95_=-1.386 p=0.169 |
| rtransversetemporal mean(SD)^a^ | 0.0157 (0.0026) | 0.0162 (0.0023) | t_92_=-1.100 p=0.274 | 0.0156 (0.0021) | 0.0152 (0.0020) | t_94_=0.874 p=0.384 | 0.0155 (0.0019) | 0.0159 (0.0022) | t_95_=-0.918 p=0.361 |
| linsula mean(SD)^a^ | 0.0166 (0.0020) | 0.0170 (0.0020) | t_92_=-0.916 p=0.362 | 0.0164 (0.0018) | 0.0168 (0.0018) | t_94_=-0.952 p=0.344 | 0.0170 (0.0015) | 0.0172 (0.0021) | t_95_=-0.421 p=0.675 |
| rinsula mean(SD)^a^ | 0.0167 (0.0018) | 0.0173 (0.0024) | t_92_=-1.327 p=0.188 | 0.0166 (0.0019) | 0.0167 (0.0020) | t_94_=-0.292 p=0.771 | 0.0171 (0.0016) | 0.0173 (0.0021) | t_95_=-0.491 p=0.625 |

^a^ two-sample t-tests between individuals with moderate / severe (any score ≥ 3) and mild (all scores < 3) negative symptoms

at follow-up. All scores are normalized by intracranial volumes.

**References**

**1.** Gaebel W, Falkai P, Weinmann S, Wobrock T. Praxisleitlinien in Psychiatrie und Psychotherapie, Band 1, Behandlungsleitlinie Schizophrenie. Steinkopff, Darmstadt: Deutsche Gesellschaft für Psychiatrie, Psychotherapie und Nervenheilkunde (DGPPN); 2006.

**2.** Kantrowitz JT *et al*. D-serine for the treatment of negative symptoms in individuals at clinical high risk of schizophrenia: a pilot, double-blind, placebo-controlled, randomised parallel group mechanistic proof-of-concept trial. *Lancet Psychiatry* May 2015;2(5):403-412.

**3.** McGlashan T, Walsh B, Woods S. The Psychosis-Risk Syndrome: Handbook for Diagnosis and Follow-up. Oxford University Press: New York, 2010.

**4.** Luders E *et al*. A curvature-based approach to estimate local gyrification on the cortical surface. *Neuroimage* Feb 2006;29(4):1224-1230.

**5.** Dahnke R, Yotter RA, Gaser C. Cortical thickness and central surface estimation. *Neuroimage* Jan 2013;65:336-348.

**6.** Spalthoff R, Gaser C, Nenadić I. Altered gyrification in schizophrenia and its relation to other morphometric markers. *Schizophr Res* Dec 2018;202:195-202.

**7.** Besteher B *et al*. Brain structural correlates of irritability: Findings in a large healthy cohort. *Hum Brain Mapp* 12 2017;38(12):6230-6238.

**8.** Desikan RS *et al.* An automated labeling system for subdividing the human cerebral cortex on MRI scans into gyral based regions of interest. *Neuroimage* Jul 2006;31(3):968-980.

**9.** Koutsouleris N *et al*. Disease prediction in the at-risk mental state for psychosis using neuroanatomical biomarkers: results from the FePsy study. *Schizophr Bull* Nov 2012;38(6):1234-1246.

**10.** Beck AT, Ward CH, Mendelson M, Mock J, Erbaugh J. An inventory for measuring depression. Arch Gen Psychiatry 1961; 4: 561-571.

**11.**  Cornblatt BA et al. Preliminary findings for two new measures of social and role functioning in the prodromal phase of schizophrenia. Schizophr Bull 2007; 33(3): 688-702.
